# Circadian Gene *BMAL1* Regulation of Cellular Senescence in Thyroid Aging

**DOI:** 10.1111/acel.70119

**Published:** 2025-05-28

**Authors:** Dandan Zong, Baihui Sun, Qiting Ye, Hongxin Cao, Haixia Guan

**Affiliations:** ^1^ Department of Endocrinology Guangdong Cardiovascular Institute, Guangdong Provincial People's Hospital, Guangdong Academy of Medical Sciences Guangzhou China; ^2^ Department of General Surgery & Guangdong Provincial Key Laboratory of Precision Medicine for Gastrointestinal Tumor Nanfang Hospital, Southern Medical University Guangzhou China; ^3^ Department of Endocrinology Guangdong Provincial People's Hospital, Guangdong Academy of Medical Sciences, Southern Medical University Guangzhou China; ^4^ Key Laboratory for Tumor Precision Medicine of Shaanxi Province and Department of Endocrinology The First Affiliated Hospital of Xi'an Jiaotong University Xi'an China

**Keywords:** BMAL1, cellular senescence, rhythm, thyroid aging

## Abstract

As global aging accelerates, the incidence of thyroid diseases, particularly hypothyroidism, is rising in the elderly. The thyroid‐stimulating hormone (TSH) levels increase in healthy elderly populations. However, whether the thyroid undergoes cellular senescence and how this relates to thyroid hormone (TH) synthesis remain unclear. To investigate the molecular and functional characteristics of thyroid aging, we performed scRNA‐seq on human thyroids from young, middle‐aged, and old groups, identifying thousands of aging‐related differentially expressed genes and revealing the early onset of aging in the middle‐aged group. As aging progresses, the expression levels of genes related to TH synthesis increase, suggesting that epithelial cells (EPI) adjust their gene expression in response to elevated TSH levels. Additionally, the senescence‐associated secretory phenotype (SASP) in EPI cells is progressively enhanced with aging. We identified a subgroup of epithelial cells (CDKN1A_EPI) characterized by reduced functionality and significantly elevated levels of cellular senescence. We found that the core circadian rhythm gene *BMAL1* (*ARNTL*) is downregulated during aging. We further validated this finding using the thyroid‐specific *Bmal1* knockout mouse model, showing that the downregulation of *Bmal1* inhibits the expression of *Nfkbia* (*NF‐κB inhibitor alpha*), thereby accelerating cellular senescence and impairing hormone synthesis. Finally, through cell line experiments and transcriptome sequencing, we confirmed that *BMAL1* knockout leads to decreased *NFKBIA* expression, promoting thyroid cellular senescence. Our study demonstrates that circadian rhythm disruption accelerates cellular senescence in the thyroid and exacerbates the decline of thyroid function, providing a novel theoretical foundation for understanding thyroid aging mechanisms and maintaining thyroid function stability.

## Graphical abstract

BMAL1 downregulation during aging reduces NFKBIA expression, accelerating thyroid cellular senescence and impairing hormone synthesis. Circadian rhythm disruption exacerbates thyroid functional decline, highlighting the BMAL1‐NFKBIA axis as a key mediator of aging‐related thyroid dysfunction and offering insights for stabilizing thyroid health in aging populations.

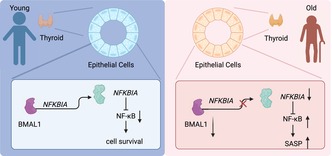

## 1 Introduction

The thyroid regulates synthesis/secretion of thyroid hormone (TH) that critically maintains energy metabolism (Mullur et al. 2014). The thyroid is composed of various cell types, with thyroid epithelial cells (EPI) being the most prevalent, tasked with synthesizing and secreting TH (Hong et al. 2023). EPI generates triiodothyronine/thyroxine (T3/T4) through thyroid peroxidase (TPO)‐catalyzed iodination of thyroglobulin (TG) tyrosine residues (Fonseca et al. 2013). Thyroid stimulating hormone (TSH), released by the pituitary gland, acts on the thyrotropin receptor (TSHR) in thyroid follicular cells, promoting the synthesis and release of TH (Fliers et al. 2014). Recent studies have identified a connection between TH dysregulation and aging, as well as age‐related diseases (Franceschi et al. 2019; Zhang et al. 2024). With the global increase in aging populations, the incidence of thyroid diseases in the elderly is rising, particularly hypothyroidism. Research indicates that TSH levels significantly increase while TH levels decrease notably in healthy elderly individuals (Aggarwal and Razvi 2013; Belden and Dunlap 2013; Boelaert 2013). Therefore, an in‐depth understanding of the molecular mechanisms of human thyroid aging is of great scientific and clinical importance.

Aging is a complex biological process involving the progressive functional decline of various mechanisms and systems (Chakravarti et al. 2021; Lopez‐Otin et al. 2013, 2022, 2023). Cellular senescence is characterized by irreversible cell cycle arrest mediated by CDKN1A/p21 and CDKN2A/p16 overexpression, accompanied by senescence‐associated β‐galactosidase (SA‐β‐gal) activation (Mijit et al. 2020). These senescent cells secrete senescence‐associated secretory phenotype (SASP) components including pro‐inflammatory factors that disrupt tissue integrity and accelerate aging (Zhao et al. 2024). Cellular senescence leads to a reduction in the number of functional cells within tissues, thereby impairing normal tissue function (Xie et al. 2023). The accumulation of senescent cells has been shown to accelerate the onset of various diseases, and their clearance can reverse aging in the organism (Faggioli et al. 2023; Lee et al. 2023; Zhang, Habiballa et al. 2022). However, the presence and role of cellular senescence in thyroid tissue remain to be explored.

Aging disrupts biological rhythms, including circadian regulation of the hypothalamic–pituitary–thyroid (HPT) axis by the suprachiasmatic nucleus, which controls TH secretion rhythms (Fliers et al. 2014). Multiple studies have reported that rhythm disruptions are high‐risk factors for thyroid diseases (Khosravipour et al. 2024; Rizza et al. 2020). Sleep deprivation and night shifts induce circadian disturbances that dysregulate TH secretion, elevating TSH and reducing TH levels while maintaining physiological ranges (Khosravipour et al. 2024). The circadian regulator BMAL1 coordinates biological rhythms, and its astrocyte‐specific deletion disrupts circadian function while triggering autonomous cellular activation (Barca‐Mayo et al. 2020; Kondratov et al. 2006; Li et al. 2020; Liang et al. 2022). Recent research has demonstrated that BMAL1 is crucial for cellular senescence (Liang et al. 2022; Zhang, Xiong et al. 2022). However, the impact of age‐related rhythm disruptions on thyroid aging remains unknown.

Despite previous studies revealing some mechanisms of thyroid aging, comprehensively understanding its changes and underlying mechanisms at the single‐cell level remains an unsolved mystery. Therefore, this study aims to systematically analyze the cellular composition and gene expression characteristics of pathologically normal thyroid tissues from different age groups through scRNA‐seq, with a particular focus on the subpopulations of epithelial cells. We divided the samples into three groups: young (18–35 years), middle‐aged (35–65 years), and elderly (> 65 years). Using mouse models and in vitro experiments, we validated and investigated the role of *BMAL1* in thyroid cellular senescence. Our study reveals the mechanism by which circadian rhythm disruption promotes thyroid cellular senescence and exacerbates functional decline, providing new potential targets and a theoretical foundation for future interventions in thyroid aging‐related diseases.

## 2 Results

### 2.1 Single‐Cell Transcriptome Profiles of the Human Thyroid in Young, Middle‐Aged, and Old Groups

**FIGURE 1:**
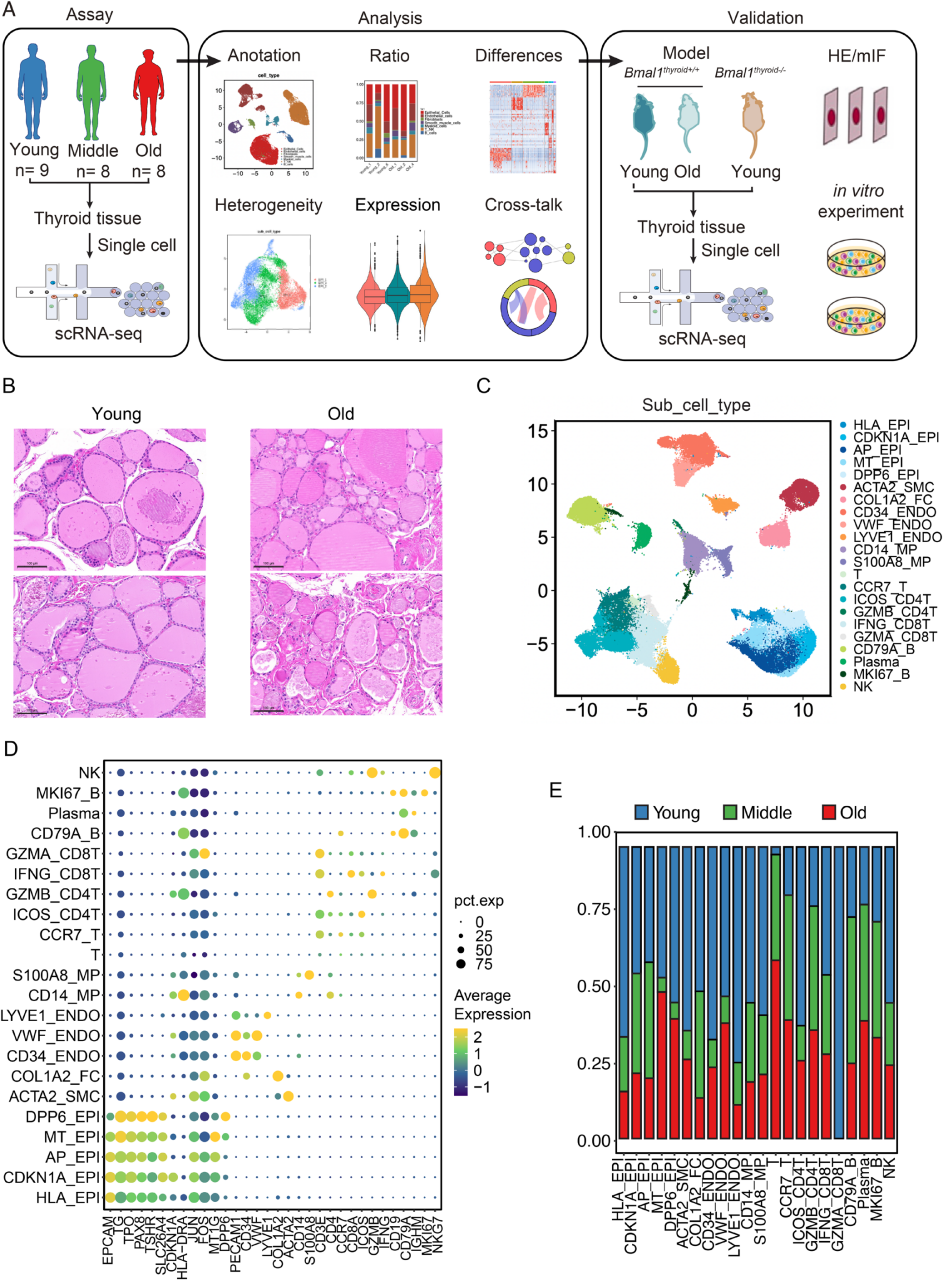
Cell type identification by single‐cell RNA‐seq analysis of human thyroid in young, middle‐aged, and old groups. (A) Flowchart overview of single‐cell RNA‐seq of human thyroid of different age groups. (B) Hematoxylin and eosin (H&E) stained sections of human thyroid from young and old donors. Scale bar, 100 μm. (C) UMAP plots showing subtypes of different age groups in human thyroid. (D) Dot plot showing the gene expression signatures of marker genes corresponding to each cell type in human thyroid. The dot size indicates the fraction of expressing cells, and the color indicates the expression level. (E) Bar plots illustrate the proportion of human thyroid for each cell type.

To determine the effects of aging on the thyroid, we collected human thyroids from individuals of various ages, utilizing both publicly available datasets and data from our own research (Figure 1A). During aging, thyroid tissue tends to show irregular follicle sizes and distribution, disorganized cell arrangement, increased cell spacing, and a looser overall structure, as previously reported (Figure 1B). We performed scRNA‐seq on 25 thyroid samples from individuals who were young (Young), middle‐aged (Middle), and old (Old) (Figure 1A and Table S1). This allowed us to identify cell‐type‐specific variations in gene expression that occur during the aging process of the thyroid. A total of 134,360 cells were included in the subsequent study (Figure S1A), with duplicate cells and cells of poor quality removed. We visualized global human thyroid cell populations by using uniform manifold approximation and projection (UMAP) (Figure S1A). Based on the expression matrix of their specific markers, we identified eight major cell types. These cell types include epithelial cells (EPI), endothelial cells (ENDO), smooth muscle cells (SMC), fibroblasts (FC), myeloid cells (MP), T cells (T), B cells (B), and natural killer cells (NK) (Figure S1A). Further, the cells were classified into 22 subtypes, which are as follows: HLA_EPI, CDKN1A_EPI, AP_EPI, MT_EPI, DDP6_EPI, ACTA2_SMC, COL1A2_FC, CD34_ENDO, VWF_ENDO, LYVE1_ENDO, CD14_MP, S100A8_MP, T, CCR7_T, ICOS_CD4T, GZMB_CD4T, IFNG_CD8T, GZMA_CD8T, CD79A_B, Plasma, MKI67_B, and NK Cells (Figure 1C). Data integration was conducted using Harmony to remove batch effects and ensure consistency and comparability (Figures 1C and S1B,C). In Figures 1D and S2A, the biomarker expression that was specific to the subtype was displayed, respectively.

Analysis of the significant difference genes for each cell type revealed unique transcriptional features and enriched pathways relevant to their distinct physical functions (Figure S2B, Tables S2 and S3). For example, the significant difference genes of epithelial cells and CDKN1A_EPI were shown to be enriched for Gene Ontology (GO) related to the “metabolism of thyroid hormone” and “reaction to endoplasmic reticulum stress.” The proportions and distribution of cell types identified using scRNA‐seq were similar in young, middle‐aged, and old donors, suggesting that the cell‐type identity of the thyroid may not alter with age (Figures 1E and S1C). Epithelial cells were mainly identified as the main cell type associated with the detection of hotspot genes linked to aging and thyroid disorders (Figure S3). This discovery emphasizes the crucial role of epithelial cells in controlling thyroid homeostasis and the aging process.

### 2.2 Gene Expression Changes in Different Cell Types During Human Thyroid Aging at Single‐Cell Resolution

**FIGURE 2:**
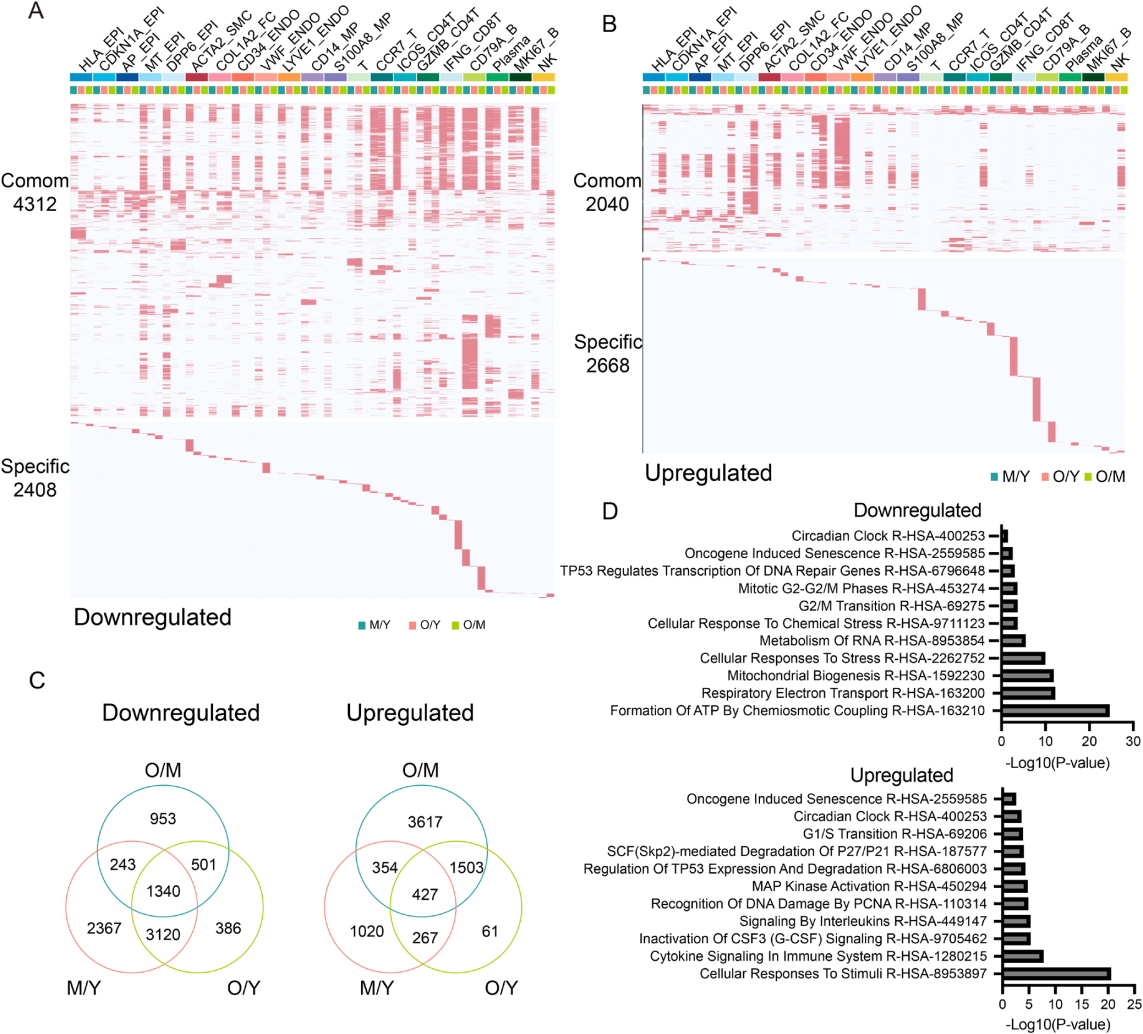
Changes in the transcriptional profiles of different cell types during human thyroid aging. (A and B) Heatmaps showing the distribution of downregulated (A) and upregulated (B) DEGs for each cell type in human thyroid between the old and young groups (O/Y), middle‐aged and young groups (M/Y), and old and middle‐aged groups (O/M). Genes not differentially expressed are in gray, and the numbers of DEGs are indicated. The upper part indicates the DEGs shared by at least two columns; the lower panel indicates the unique DEGs of each column. The numbers of genes are annotated on the plots. (C) Venn plots showing the number of shared downregulated (right) and upregulated (left) DEGs between different groups of human thyroid samples. (D) Representative shared Reactome terms of downregulated (upper) and upregulated (lower) common DEGs of different age groups.

Aging usually leads to increased transcriptional noise (Angelidis et al. 2019; Salzer et al. 2018). By calculating the age‐relevant coefficients of variation to assess levels of variant transcriptional noise, we found higher levels of aging‐accumulated transcriptional noise in epithelial cells and myeloid cells than in other cell types (Figure S4A), suggesting a higher vulnerability of epithelial cells and myeloid cells to age‐related stress compared to other cell types. To further explore the mechanism of thyroid aging at the cellular level, we separated the dataset into three groups: young (Y), middle‐aged (M), and old (O). Then, we compared the gene expression patterns of individual subtypes between the three groups. This analysis revealed thousands of differentially expressed genes (DEGs) in at least one cell type of the human thyroid during aging (|avg_logFC| > 0.25 and *p*_val_adj < 0.05) (Figures 2A,B and S4B). We identified 6720 down‐regulated DEGs between the young and middle‐aged (M/Y) groups, the middle‐aged and old (O/M) groups, and the young and old (O/Y) groups, respectively. In comparison, 4708 up‐regulated DEGs were identified in the M/Y, O/M, and O/Y groups, respectively (Figure 2A,B). We categorized the differential genes into common and specific groups based on their presence in at least two groups. It was observed that many of the O/Y DEGs were already identified in the M/Y group (Figure 2A,B). We merged the DEGs from the M/Y and O/M groups, denoted as M/Y & O/M, and compared them with the O/Y group. We observed that nearly all DEGs from the O/Y group were present in the M/Y & O/M groups (Figure S4C). Principal component analysis of DEGs further revealed that middle‐aged individuals were closer to old individuals at the transcriptomic level (Figure S4D,E), indicating the early onset of human thyroid aging. To shed light on the gene expression and functional changes that occur in the aging thyroid, we overlapped the M/Y, O/M, and O/Y groups. We discovered that 1340 down‐regulated DEGs and 427 up‐regulated DEGs showed consistent changes in gene expression during aging (Figure 2C). The functional enrichment analysis of down‐regulated DEGs revealed associations with signaling pathways related to mitochondrial function and stress response pathways. In contrast, up‐regulated DEGs were connected with cellular stress and cytokine signaling pathways (Figure 2D). These findings are in line with a substantial body of aging research literature that has consistently reported similar functional associations (Lopez‐Otin et al. 2022; F. L. Zhang et al. 2022).

### 2.3 Age‐Related Changes of Thyroid Function in Epithelial Cells

To understand the changes in thyroid function with age, we examined the expression levels of specific genes associated with thyroid cells derived from individuals of varying ages. Our findings revealed that the expression of these genes varies with age. Specifically, as individuals aged, the expression levels of *TG*, *TPO*, *TSHR*, and *PAX8* significantly increased (Figure S5A). This suggests that the synthesis functions of TH change with age. Immunohistochemistry (IHC) was used to further validate that the protein levels of these genes increase with age (Figure S5B). This indicates that the elevated expression of TH synthesis‐related genes in thyroid cells may be a response to the increased levels of TSH induced by aging. By analyzing scRNA‐seq, the study also observed age‐related expression changes of these genes in different thyroid cell subpopulations. For instance, *TG* expression in CDKN1A_EPI, AP_EPI, and DDP6_EPI increased with age, while *TPO* expression in HLA_EPI, MT_EPI, and DDP6_EPI also showed an increase. *TSHR* expression in HLA_EPI increased with age, and *PAX8* expression was highest in middle age among HLA_EPI, CDKN1A_EPI, AP_EPI, and DDP6_EPI subpopulations (Figure S5C). These findings suggest that the higher prevalence of thyroid disease in the elderly may be attributed to age‐related changes in thyroid function.

### 2.4 Accumulation of Senescent Thyroid Epithelial Cells With Reduced Hormone Synthesis in the Elderly

**FIGURE 3:**
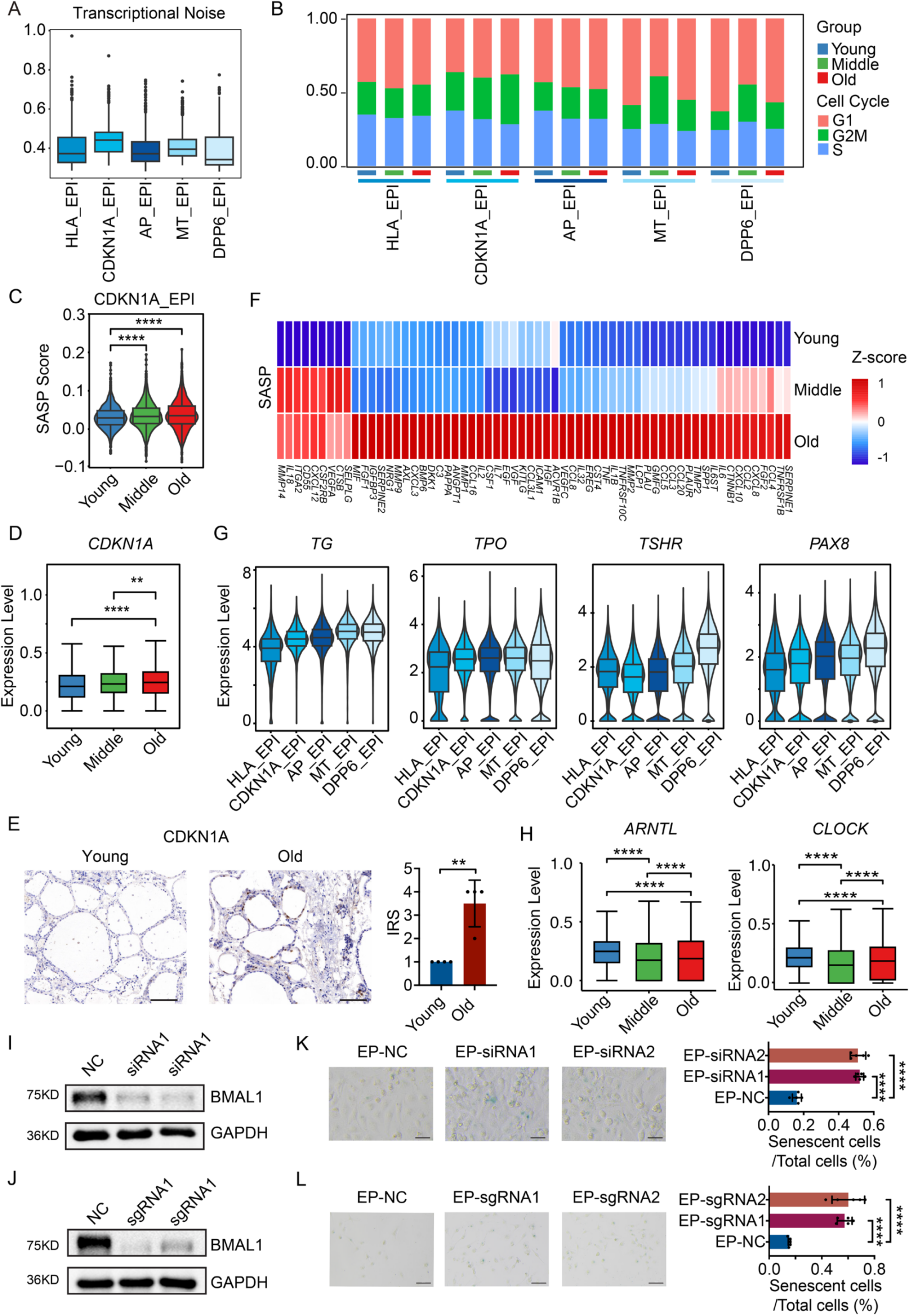
Cellular senescence in human thyroid. (A) Transcriptional noise of subtype cells in thyroid epithelial cells. (B) Ratio plot of cell cycle in thyroid epithelial subtype cells of young, middle‐aged, and old groups. (C) The SASP gene set scores in CDKN1A_EPI cells of young, middle‐aged, and old groups. SASP, senescence‐associated secretory phenotype. (D) Gene expression level of *CDKN1A* in thyroid epithelial cells of young, middle‐aged, and old groups. (E) Immunohistochemical analysis of CDKN1A protein expression levels in thyroid tissues from young and aged groups, showing representative images (scale bar, 100 μm) and statistical results of the Remmele immunoreactive score (IRS). (F) Heatmaps showing gene expression level of gene set SASP in thyroid epithelial subtype CDKN1A_EPI cells of young, middle‐aged, and old groups. SASP, senescence‐associated secretory phenotype. (G) Gene expression levels of *TG*, *TPO*, *TSHR*, and *PAX8* in thyroid epithelial subtype cells of young, middle‐aged, and old groups. (H) Gene expression levels of *ARNTL* and *CLOCK* in thyroid epithelial cells of young, middle‐aged, and old groups. (I) Western blot analysis of BMAL1 and GAPDH in control and BMAL1 knockdown cell lines. (J) Western blot analysis of BMAL1 and GAPDH in control and BMAL1 knockout cell lines. (K) Representative images and quantitative analysis of SA‐β‐gal staining are shown for cell lines in the control and *BMAL1* knockdown cell line. Scale bar, 100 μm. (L) Representative images and quantitative analysis of SA‐β‐gal staining are shown for cell lines in the control and *BMAL1* knockout cell line. Scale bar, 100 μm. **p < 0.01, ****p < 0.0001.

We noted that both up‐ and down‐regulated genes were notably enriched in the gene sets associated with “cellular senescence” (Figure 2D). This suggests that cellular senescence is closely related to thyroid aging. We then calculated the transcriptional noise for epithelial cell subgroups and found that the CDKN1A_EPI group exhibited high levels (Figure 3A). Genes specifically highly expressed in the CDKN1A_EPI group were significantly enriched in pathways related to endoplasmic reticulum stress (Figure S2B), which is one of the characteristics of senescent cells (Lopez‐Otin et al. 2022). Cellular senescence typically leads to cell cycle arrest at the G1 phase (Kuilman et al. 2010). We analyzed the cell cycle distribution of different cell subgroups and found that the proportion of cells in the G1 phases in the CDKN1A_EPI group increased with age (Figure 3B). These findings indicate that the CDKN1A_EPI exhibits characteristics of cellular senescence. The SASP gene set (SenMayo panel, Table S4) (Saul et al. 2022) scores across different age groups revealed a significant age‐related increase in the CDKN1A_EPI group (Figure 3C). We also calculated the expression of *CDKN1A*, a marker of cellular senescence, and found that its gene expression increases during aging (Figure 3D). Immunohistochemical results further confirmed the increased expression of CDKN1A, CDKN2A, and SA‐β‐gal in the elderly (Figures 3E and S6). Out of the 125 genes within the SenMayo panel, most were upregulated during aging in CDKN1A_EPI cells (Figure 3F). For example, genes such as *MIF*, *IL6*, and *IL1B*, which promote chronic inflammation, show increased expression with age, consistent with the chronic inflammatory state observed in the elderly. These findings suggest the presence of senescent cells in the thyroid. The genes related to TH synthesis, *TG*, *TPO, TSHR*, and *PAX8*, show significantly lower expression levels in the CDKN1A_EPI group compared to MT_EPI, AP_EPI, and DDP6_EPI (Figure 3G). This indicates that the hormone synthesis function of CDKN1A_EPI cells is reduced. In summary, there are senescent cells with reduced hormone synthesis function in the thyroid, and these cells accumulate in the elderly.

### 2.5 Thyroid Cell‐Specific Knockout of *BMAL1* Promotes Cellular Senescence

We noted that both differentially expressed genes were significantly enriched in circadian rhythm gene sets (Figure 2D), suggesting a relationship between thyroid aging and circadian rhythms. As age increases, significant changes in biological rhythms often occur, and the thyroid is also regulated by circadian rhythms. However, *ARNTL/BMAL1* (Brain and Muscle ARNT‐Like 1) and *CLOCK* (Circadian Locomotor Output Cycles Kaput), as key genes in circadian rhythm regulation, showed decreased expression levels with age (Figure 3H), suggesting a decline in the thyroid's ability to regulate biological rhythms during aging, leading to circadian rhythm disruption. We hypothesized that the downregulation of *BMAL1* expression in thyroid cells promotes cellular senescence. To test this hypothesis, we used the normal thyroid cell line HTori‐3.1 and used siRNA and CRISPR/Cas9‐sgRNA technology to knock down or knock out *BMAL1* and similarly observed an increase in cellular senescence (Figures 3I–L and S7).

**FIGURE 4:**
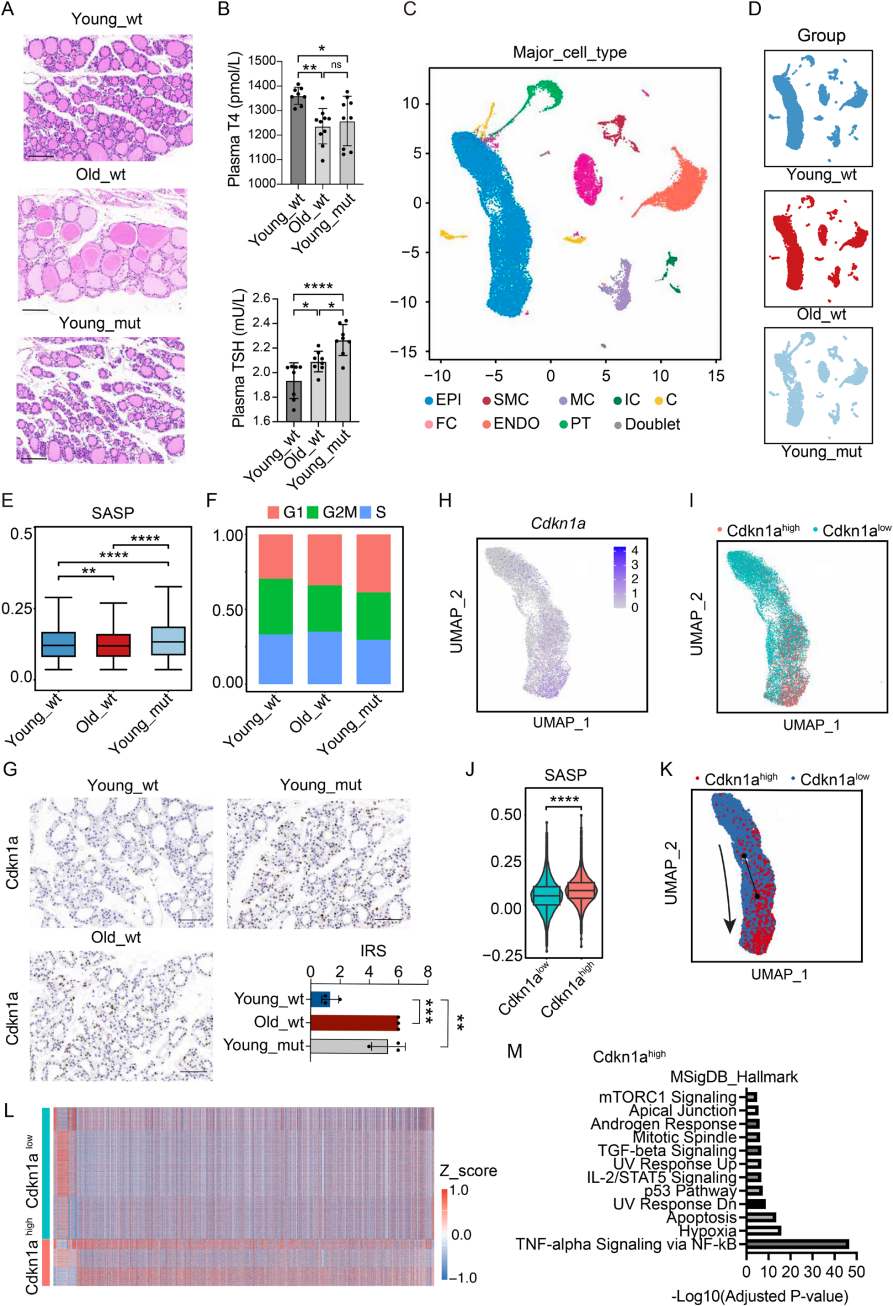
Deletion of *Bmal1* induced cellular senescence. (A) Hematoxylin and eosin (H&E) stained sections of mouse thyroid from young wild‐type mice (Young_wt), old wild‐type mice (Old_wt), and thyroid‐specific deletion of *Bmal1* in young mice (Young_mut). (B) Bar plot showing detection of thyroid‐stimulating hormone (TSH) and thyroid hormone (T4) levels in mouse serum. (C) UMAP plots showing cell types of different groups in mouse thyroid. C, C cells; ENDO, endothelial cells; EPI, epithelial cells; FC, fibroblasts; IC, immune cells; MC, myeloid cells; PT, parathyroid cells; SMC, smooth muscle cells. (D) UMAP plots showing cell distribution of different groups in mouse thyroid. (E) The SASP gene set scores in thyroid epithelial cells from young wild‐type mice, old wild‐type mice, and thyroid‐specific deletion of *Bmal1* in young mice. SASP, senescence‐associated secretory phenotype. (F) Ratio plot of cell cycle in thyroid epithelial cells from young wild‐type mice, old wild‐type mice, and thyroid‐specific deletion of *Bmal1* in young mice. (G) Immunohistochemical analysis of Cdkn1a protein expression levels in thyroid tissues from Young_wt, Young_mut, and Old_wt groups, showing representative images (scale bar, 100 μm) and statistical results of the Remmele immunoreactive score (IRS). (H) UMAP plots showing the cell distribution of *Cdkn1a* expression level in mouse thyroid epithelial cells. The color indicates the expression level. (I) UMAP plots showing the cell distribution of Cdkn1a^high^ and Cdkn1a^low^ in mouse thyroid epithelial cells. The color indicates the expression level. (J) Gene expression level of gene set SASP in Cdkn1a^high^ and Cdkn1a^low^ cells. SASP, senescence‐associated secretory phenotype. (K) Pseudotemporal cell ordering of all the epidermal cell types along differentiation trajectories. Cells are colored by cell type . (L) Heatmaps showing the DEGs between Cdkn1a^high^ and Cdkn1a^low^. The color indicates the DEGs expression level. (M) Representative terms for genes significantly upregulated in Cdkn1a^high^ cells. *p < 0.05, **p < 0.01, ***p < 0.001, ****p < 0.0001.

To further validate that the decline in *Bmal1* expression promotes cellular senescence, we constructed a thyroid‐specific *Bmal1* knockout mouse model and observed uneven follicular structures in young wild‐type mice (Young_wt), old wild‐type mice (Old_wt), and thyroid‐specific deletion of *Bmal1* in young mice (Young_mut) (Figures 4A and S8). We collected serum samples from Young_wt, Old_wt, and Young_mut mice, all maintained under identical light–dark cycles (12 h light/12 h dark). We measured the levels of T4 and TSH and found that *Bmal1* knockout and aging led to decreased T4 levels and increased TSH levels (Figure 4B). This indicates that circadian rhythm disruption leads to abnormal hormone synthesis. We collected thyroid tissues from Young_wt (*n* = 4), Old_wt (*n* = 4), and Young_mut (*n* = 4) under identical light–dark cycles (12 h light/12 h dark) for scRNA‐seq. We screened for high‐quality single‐cell transcriptome data and performed dimensionality reduction and clustering. Based on known markers, we annotated the resulting cell clusters and identified a total of eight cell types in mice: epithelial cells (EPI), endothelial cells (ENDO), fibroblasts (FC), smooth muscle cells (SMC), myeloid cells (MC), immune cells (IC), parathyroid cells (PT), and C cells (C), reflecting the specific characteristics of mouse thyroid tissue (Figure 4C,D and Table S5). The expression patterns of cell‐specific marker genes further confirmed the accuracy of cell annotation (Figure S9A,B). Consistent with our findings in human thyroids, the expression levels of TH synthesis‐related genes *Tg* and *Tshr* were elevated in thyroid epithelial cells from the *Bmal1* knockout mice (Figure S9C). In response to elevated TSH, thyroid cells compensatorily increase the expression of hormone synthesis‐related genes.

By calculating gene set expression, we found that thyroid cellular senescence scores significantly increased in the aged group and gene knockout group (Figure 4E). We analyzed the cell cycle distribution of different groups and found that the G1 phase proportion of cells increased in the aged group and gene knockout group (Figure 4F). We also confirmed the increased expression of Cdkn1a, Cdkn2a, and SA‐β‐gal in the gene knockout group by immunohistochemical analysis (Figures 4G and S10). These results indicate that, consistent with findings in human thyroid aging, thyroid cellular senescence occurs in aged mice. In thyroid cells with *Bmal1* knockout, cellular senescence was also induced.

To investigate the molecular mechanism by which BMAL1 regulates cellular senescence, we analyzed the expression levels of *Cdkn1a* in mouse thyroid cells. We defined cells with high *Cdkn1a* expression as Cdkn1a^high^ and the remaining cells as Cdkn1a^low^ (Figure 4H,I). The cellular senescence score is significantly higher in the Cdkn1a^high^ group (Figure 4J). Pseudotime analysis of mouse thyroid cells revealed a differentiation trajectory from Cdkn1a^low^ to Cdkn1a^high^ (Figure 4K). Heatmap analysis of differentially expressed genes between Cdkn1a^high^ and Cdkn1a^low^ cells showed distinct transcriptomic features (Figure 4L and Tables S6). Functional enrichment analysis of genes highly expressed in Cdkn1a^high^ cells significantly enriched NF‐κB‐related pathways (Figure 4M).

### 2.6 *BMAL1* Downregulation Drives Cellular Senescence by Suppressing *NFKBIA* Expression

**FIGURE 5:**
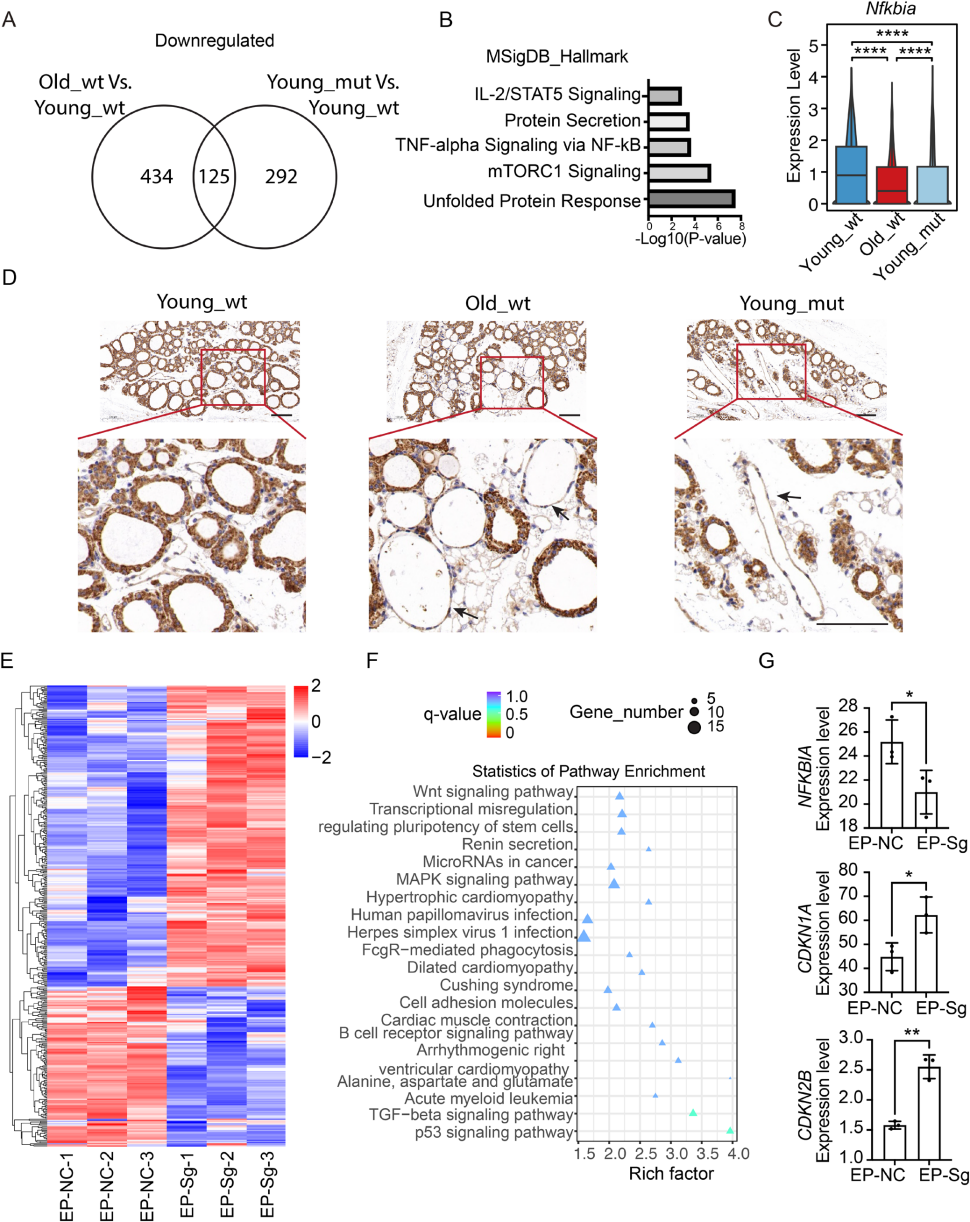
*NFKBIA* depression of deletion *BMAL1*‐induced cellular senescence. (A) Venn plots showing the number of shared downregulated DEGs between Old_wt vs. Young_wt and Young_mut vs. Young_wt. (B) Representative terms of shared DEGs between Old_wt vs. Young_wt and Young_mut vs. Young_wt. (C) Gene expression level of *Nfkbia* in thyroid epithelial cells from young wild‐type mice, old wild‐type mice, and thyroid‐specific deletion of *Bmal1* in young mice. (D) Immunohistochemical assay of protein expression level of Nfkbia in thyroid tissues from young wild‐type mice, old wild‐type mice, and thyroid‐specific deletion of *Bmal1* in young mice. Scale bar, 100 μm. (E) Heatmaps showing different expression genes in control and *BMAL1* knockout cell lines. (F) Representative terms of different expression genes between the control and *BMAL1* knockout cell lines. (G) The expression of *NFKBIA*, *CDKN1A*, and *CDKN2B* in control and *BMAL1* knockout cell lines. *p < 0.05, *p < 0.01, ***p < 0.001.

We merged the downregulated genes from the old and young groups, denoted as Old_wt vs. Young_wt, and the downregulated genes from the old and gene knockout groups, denoted as Young_mut vs. Young_wt. We compared and observed that 125 genes are common in the old and gene knockout groups (Figure 5A). Functional enrichment analysis of common genes significantly enriched NF‐κB‐related pathways (Figure 5B). Compared with the young group, the expression levels of *Nfkbia* also decreased in the old and gene knockout groups (Figure 5C). Immunohistochemical examination of mouse thyroids revealed that some cells in aged mice and gene knockout mice exhibited noticeably low Nfkbia expression (Figure 5D, arrows indicating representative cells) alongside concurrent upregulation of phosphorylated p65 (p‐p65) (Figure S11A), thereby confirming NF‐κB pathway activation in these populations. Furthermore, we extended this analysis to human thyroid tissues from elderly subjects, with parallel NF‐κB pathway activation (p‐p65 accumulation) observed in these samples (Figure S11B).

To further explore the relationship between BMAL1 and cellular senescence, we performed RNA‐seq sequencing on the *BMAL1* knockout and the control group, identified 143 downregulated and 277 upregulated differentially expressed genes (Figure 5E). These genes were significantly enriched in the “TP53 signaling pathway” and the “TGF‐beta signaling pathway” (Figure 5F), both known to interact with NF‐κB signaling during cellular senescence (Aging Biomarker et al. 2023; Mijit et al. 2020; Xie et al. 2023). In the knockout group, *NFKBIA* expression was downregulated, while the expression of the cellular senescence marker *CDKNIA* was increased (Figure 5G). Previous studies suggest NF‐κB suppresses BMAL1 expression (Shen et al. 2021). To explore potential feedback regulation, we treated HTori‐3 cells with LPS to induce senescence (Figure S12A). LPS activated NF‐κB (via p‐p65 phosphorylation) but did not reduce BMAL1 levels (Figure S12B,C), indicating that BMAL1 downregulation likely initiates senescence upstream of NF‐κB activation, rather than resulting from it. These results indicate that *BMAL1* knockout induces cellular senescence by inhibiting *NFKBIA* expression.

### 2.7 Attenuation of Intercellular Communication Networks During Thyroid Aging

**FIGURE 6:**
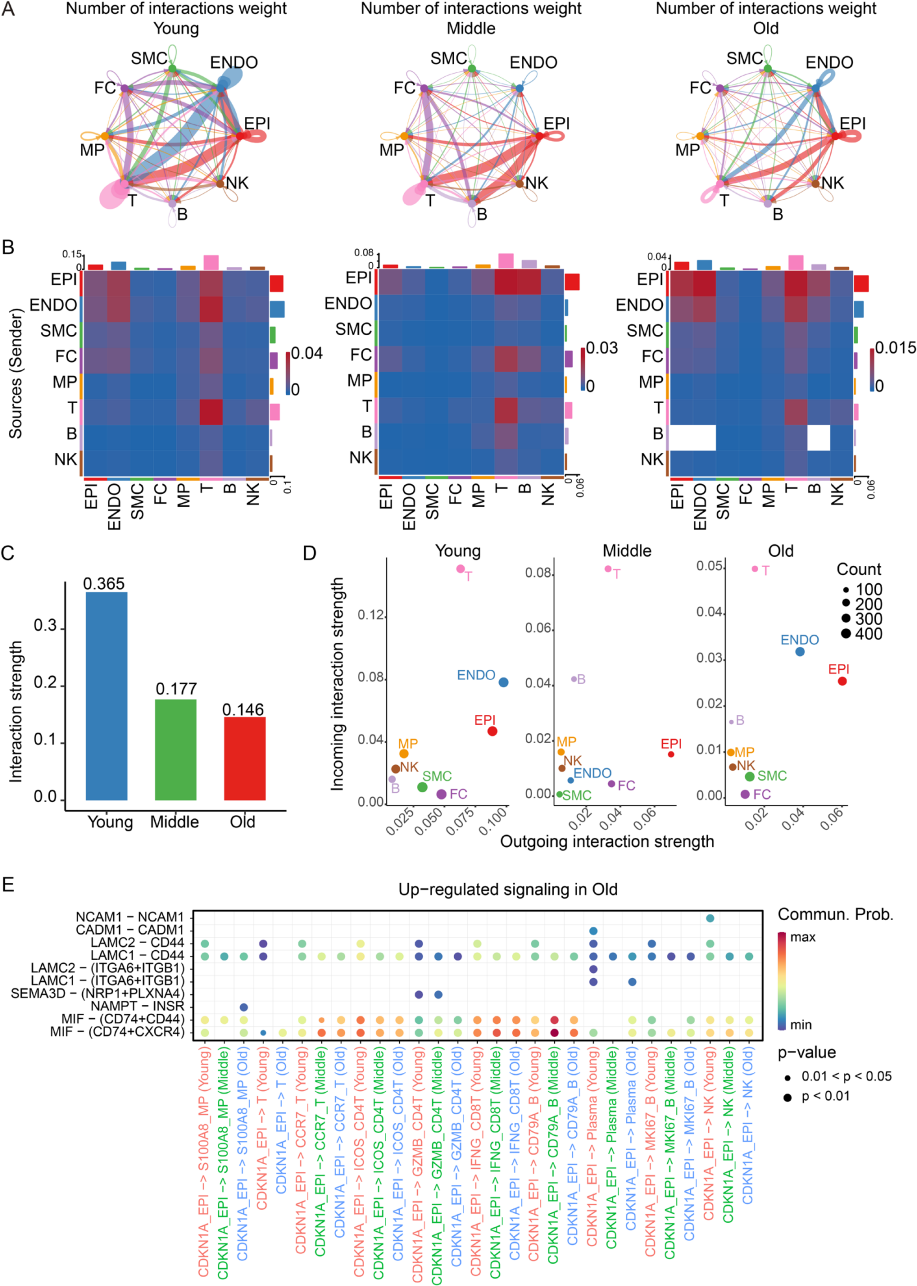
Interactions between thyroid cells weaken during aging. (A) The number of interactions weight between cell types during thyroid aging. The color key corresponds to cell type. Line thickness represents interaction strength. (B) Heatmaps showing the interaction strength between cell types during thyroid aging. The color key corresponds to interaction strength. (C) Bar plot showing the interaction strength during thyroid aging. (D) Dot plot showing the interaction strength of major cell types during thyroid aging. (E) Changes of selected ligand‐receptor interactions between CDKN1A_EPI and immune cell types during thyroid aging. Dot size indicates the *p*‐value. The color key corresponds to the mean expression level (Log2) of each interacting module.

The process of aging results in substantial modifications in the exchange of information between cells. Cell interactions in different organs exhibit organ‐specific properties, resulting in varying patterns of interaction. To study the effects of age‐related changes on communication between cells in the thyroid, we utilized CellChat analysis to analyze the networks of interactions among different cell populations. The findings of our study indicate that the number and strength of connections between cells in the thyroid diminish as an individual ages (Figure 6A). We quantified the interaction strength of each cell subpopulation as both signal sources and receivers across different age groups. The old group exhibited a significantly decreased interaction strength compared to the young and middle‐aged groups, regardless of whether they were signal sources or receivers. This suggests that intercellular communication is significantly weakened by the aging process (Figure 6B). To further quantify this trend, we analyzed the overall interaction strength across different age groups and observed that the young group exhibited the highest interaction strength, followed by the middle‐aged group, with the old group showing the lowest interaction strength (Figure 6C). The reduction in interactions suggests that signal transduction, metabolic regulation, and other cellular functions may be impaired, potentially affecting the homeostasis and functionality of thyroid tissue.

Subsequently, we evaluated the impact of distinct subsets of cells on the magnitude of interaction in different age cohorts. Endothelial cells exerted significant control over T cells in youthful tissues. Epithelial cells have been identified as the key regulators of immune cells in middle‐aged tissues. During the old period, epithelial cells exhibit increased activity in the regulation of both endothelial cells and T cells (Figure 6D). The observed change indicates that epithelial cells progressively assume the role of the main controlling cells during the aging of the thyroid. In addition, we noticed a decrease in the diversity and strength of signaling patterns as age increased. It is worth mentioning that there was a considerable decrease in signals related to immune cells, as seen in Figure S13. The decrease in connections with immune cells suggests impaired signaling pathways, which could impact the regular functioning of thyroid cells and raise the likelihood of thyroid illnesses.

During aging, CDKN1A_EPI cells were characterized by a high interaction with immune cells, with the macrophage migration inhibitory factor (MIF) pathways significantly enriched (Figure 6E). MIF is a cytokine that regulates inflammatory responses and immune function and is also a component of the SASP (Nelson et al. 2012; Wu et al. 2024). We observed that MIF was highly expressed in aged thyroid tissue (Figure 3F) and showed enhanced interactions with immune cells. These findings suggest that during thyroid aging, the accumulation of SASP is accompanied by localized chronic inflammation, which not only affects the normal function of thyroid cells but also accelerates their aging process.

## 3 Discussion

In this study, we collected healthy and adjacent non‐cancerous tissues as controls and performed a comprehensive analysis of the single‐cell transcriptome of human thyroid tissues. Through the analysis of scRNA‐seq data from young, middle‐aged, and elderly thyroid samples, we provided a comprehensive overview of the human thyroid aging process. This dataset revealed thousands of cell‐type‐specific differentially expressed genes (DEGs), highlighting the molecular changes occurring during thyroid aging. We thoroughly compared age‐related changes in the thyroid, noting that signs of aging begin to appear in middle‐aged individuals. The characteristics associated with thyroid aging are related to known aging mechanisms, including endoplasmic reticulum stress, mitochondrial dysfunction, and cellular stress (Aging Biomarker et al. 2023). Under normal physiological conditions, the synthesis of TH is regulated by physiological cycles. However, with advancing age, the function of the endogenous biological clock (such as the suprachiasmatic nucleus of the hypothalamus, SCN) declines (Kuintzle et al. 2017). This can lead to disruptions in circadian rhythm (Yang et al. 2016), subsequently affecting the secretion and regulation of TH. Studies have found that sleep deprivation and night shift work increase the likelihood of thyroid dysfunction and are risk factors for thyroid tumors (Chen et al. 2021; Fu et al. 2023; Luo et al. 2023; Rizza et al. 2020; Zhang et al. 2021).

scRNA‐seq revealed a CDKN1A‐high epithelial subpopulation (CDKN1A_EPI) exhibiting enhanced SASP signaling relative to other subtypes. These cells showed downregulated TH synthesis genes, indicating compromised hormonal capacity, while distinct thyroid cell subtypes demonstrated differential homeostatic regulation. Our findings confirm the presence of senescence in the thyroid and its accumulation with age. Notably, cells with high *CDKN1A* expression also exhibited upregulated SASP‐related genes, along with downregulated expression of *TG* and *TPO*, which is consistent with our findings in human thyroid data that suggest a decline in TH synthesis capability in senescent thyroid cells. In summary, these results indicate that due to circadian rhythm disruption in elderly individuals, the body compensates for the functional insufficiency of senescent thyroid cells by promoting the upregulation of TH synthesis‐related genes in other cells, thereby maintaining stable hormone levels.

To verify that circadian rhythm disruption leads to the accumulation of senescent cells, we compared thyroid‐specific *Bmal1* knockout mice with controls and observed an increase in SASP signaling. The knockout of *Bmal1* in mice led to decreased levels of T4 and increased TSH levels, indicating that rhythm disruption affects TH synthesis. However, we found that the expression of *Tg* genes, which are involved in TH synthesis, was increased in the knockout of *Bmal1* in mice. These results suggest that thyroid cells upregulate TH synthesis‐related genes as a compensatory response to the elevated TSH levels caused by rhythm disruption. Thus, compensatory upregulation of TH‐synthesis genes in non‐senescent cells preserves homeostasis during aging.

The expression of *BMAL1* in epithelial cells regulates the downregulation of *NFKBIA* expression. Previous studies have demonstrated that reduced NFKBIA expression accelerates cellular senescence (Kolesnichenko et al. 2021). The ChIP‐seq data from ENCODE (https://www.encodeproject.org/) indicate that there are binding sites for *BMAL1* in the promoter region of the *NFKBIA* gene, which provides evidence for how BMAL1 downregulation causes a reduction in *NFKBIA* expression. In the circadian rhythm‐regulated thyroid, aging leads to a weakening of the biological clock's control, resulting in decreased BMAL1 expression, promoting cellular senescence, and inhibiting the synthesis and secretion of TH. Therefore, circadian rhythm disruption may be a driving factor in the disturbance of homeostasis. The relationship between circadian rhythm disruption and cellular senescence, leading to thyroid dysfunction, not only has significant clinical implications but also contributes to the advancement of scientific research in this field. Research in this area could lead to new diagnostic and therapeutic approaches, improve patient health, and deepen our understanding of circadian rhythms and the endocrine system.

## 4 Method Details

### 4.1 Human Subjects

A total of seven human normal thyroid samples were collected from individuals who were scheduled to undergo surgical treatment for thyroid nodules and where there was no history of thyroid disease in their families (Table S1). They did not receive hormone medicines or undergo radiotherapy or chemotherapy. These samples were subject to the following exclusion criteria: (i)thyroid cancer or another malignant condition; (ii) thyroid malfunction due to exposure to radioactive iodine treatment, antithyroid medication, or TH replacement; or (iii) systemic autoimmune disease, acute inflammatory disease, or pregnancy. At 08:00 in the morning before surgery, serum samples were collected to assess thyroid functions (TSH, FT4, FT3) and thyroid autoantibodies (TgAb, TPOAb) using the electrochemiluminescence method on an Architect i2000SR machine from Abbott Laboratories, USA. The additional data of normal participant samples were downloaded from publicly available datasets, including GSE182416 (*n* = 7), GSE193581 (*n* = 6), GSE134355 (*n* = 2), HRA000686 (*n* = 1), GSE191288 (*n* = 1), and GSE241184 (*n* = 1). The sample collection was approved by the Ethics Committee of the Guangdong Provincial People's Hospital, and all participants provided informed consent (KY‐Q‐2022‐305‐01).

### 4.2 Mouse Subjects

The animal tests followed conventional protocols and were conducted under anesthesia using intraperitoneal injection of pentobarbital. The procedures were approved by the Department of Laboratory Animals of the Guangdong Provincial People's Hospital (KY‐Q‐2022‐305‐1). *Bmal1* floxed animals (B6.129S4(Cg)Arntl^tm1Weit^/J, stock number 007668) and *TPO‐Cre* mice (B6.FVB(Cg)‐Tg(TPO‐cre)1Shk/J, stock number 037095) were purchased from the Jackson Laboratory on a C57bl6/J background and have been intercrossed to generate *Bmal1* ^*Thyroid−/−*^(*TPO‐Cre+*; *Bmal1* ^*flox/flox*^), which lacks the *Bmal1* gene in the thyroid epithelial cells, and *Bmal1* ^*Thyroid+/+*^ (*TPO‐Cre−*; *Bmal1* ^*flox/flox*^) that served as control mice with an intact *Bmal1* gene in all cell types. Genomic DNA was extracted from mouse toe tissues, amplified by PCR, and genotyped via agarose gel electrophoresis analysis. All animals were raised in a clean room with 12‐h of light and 12‐h of darkness, with free access to food and water. Animals were housed in the same environment to minimize potential variations in estrous cycles caused by external factors. For the daytime culling, mice were exposed to a light source to ensure proper visualization during the procedure. For nighttime culling, red lights were used in the room to avoid disrupting the natural darkness cycle of the mice. First, to ensure that the circadian rhythm of sampling was consistent across groups, we standardized sampling at 8:00 am. Thyroid tissues were obtained from *Bmal1* ^*Thyroid+/+*^ mice, young wild‐type mice (3–4 months, Young_wt), and older *Bmal1* ^*Thyroid+/+*^ mice (18–22 months, Old_wt), as well as from *Bmal1* ^*Thyroid−/−*^ mice (3–4 months, Young_mut). Following cardiac perfusion, the thyroid tissues were extracted and processed into single‐cell suspensions. The remaining samples underwent OCT processing and paraffin embedding. Mouse serum samples were stored at −80°C for subsequent hormone level measurement using an ELISA kit.

### 4.3 Single Cell Isolation

Human and mouse thyroid tissues were preserved in Miltenyi tissue storage solution in an ice bath and transferred to the laboratory within 2 h. Each thyroid sample was divided into aliquots; half was immersed in 4% paraformaldehyde (PFA) for paraffin embedding. Another tissue was washed with PBS (0.04% BSA) and cut into 0.1‐cm^3^ sections in 2 mg/mL IV collagenase. The tube containing IV collagenase and tissues was then oscillated in a water bath at 37°C for 20 min. After centrifugation at 1200 rpm for 5 min, the supernatant was discarded and the tissues were resuspended in 0.5% trypsin. The tissues in 0.5% trypsin were further oscillated in a water bath at 37°C for 10 min. Digestion was stopped with DMEM containing 10% BSA and filtered through a 40‐μm cell strainer (Millipore). Subsequently, cells were incubated in red blood cell lysis buffer for 10 min, then centrifuged and resuspended in 100–200 μL PBS containing 0.04% BSA. Overall cell viability, confirmed by trypan blue exclusion (above 85%), resulted in single‐cell suspensions, counted using a hemocytometer, and the concentration was adjusted to 700–1200 cells/μL.

### 4.4 Single‐Cell RNA‐Seq Library Preparation and Sequencing

Single‐cell transcriptome libraries were prepared according to the Chromium Next GEM Single Cell 3′ Reagent Kit v3.1 (10X Genomics). Cell suspensions were loaded onto a chromium single‐cell chip along with reverse transcription (RT) master mix and 3′ gel beads. After the generation of single‐cell gel beads in emulsion (GEMs), RT was performed using a C1000 Touch Thermal Cycler (Bio‐Rad) using the manufacturer's standard parameters. cDNA was amplified and purified with SPRIselect beads (Beckman Coulter). Single‐cell libraries were then constructed following fragmentation, end repair, poly‐A tailing, adaptor ligation, and size selection. The scRNA‐seq libraries were generated with one sample index for each sample and sequenced on the Illumina NovaSeq 6000 platform.

### 4.5 scRNA‐Seq Primary Data Processing

Cell Ranger v7.0 was used to demultiplex the FASTQ reads, align them to the GRCh38 human or GRCm38 mouse transcriptome, and extract their “cell” and “UMI” barcodes. The output of this pipeline is a digital gene expression (DGE) matrix, which records the number of UMIs for each gene that are associated with each cell barcode. Next, we created the Seurat objects for DGE matrices of three batches using Seurat4.0, and then merged them into one Seurat object. Cells with fewer than 200 genes, more than 6000 genes, and a percentage of mitochondrial genes greater than 10% were removed, and genes expressed in fewer than 10 cells were removed. Gene expression was normalized by “LogNormalize” in Seurat4.0. The output was the recovered gene expression matrix, which was used for downstream analysis.

### 4.6 Removal of Doublets and Data Integration

We applied DoubletFinder to identify potential doublets using default parameters to calculate the doublet score for each single cell, along with a threshold based on a bimodal distribution. The expected doublet rate was set to 0.05, and cells predicted to be doublets or with a doublet score greater than 0.25 were filtered out. A total of 25 scRNA‐seq datasets representing multiple ages were included in this data collection. In addition, we have included in‐house data from 7 thyroid samples from persons with no family history of thyroid disease undergoing nodule surgery, aimed at increasing the number of samples with normal thyroid for further analysis. Following this, to integrate cells from different individuals into a shared space for downstream analysis, batch effect correction was performed using the Harmony algorithm, applying the regression parameter “orig.ident.” This ensured consistent clustering and improved comparability across samples.

### 4.7 Clustering and Identification of Cell Types

All clustering analyses were conducted following the Seurat4.0 integrated tutorial. Variable genes were identified with the FindVariableFeatures function, and 2000 variable genes were selected for subsequent analysis. The first 20 principal components were used for principal component analysis (PCA). Use the Harmony function to remove batch effects on the data. Clustering was performed using the FindClusters function, which works on a k‐nearest neighbor graph model with a resolution of 1.0, and displayed in UMAP/t‐distributed stochastic neighbor embedding plots.

To identify DEGs, we used the Seurat FindMarkers function based on a Wilcox likelihood‐ratio test with default parameters and selected the genes expressed in more than 25% of the cells in a cluster and with an average log2(fold change) value greater than 2 as DEGs. For the cell type annotation of each cluster, we combined the expression of canonical markers found in the DEGs with knowledge from the literature and displayed the expression of markers of each cell type with FeaturePlot and violin plots that were generated with the Seurat FeaturePlot/VlnPlot function. The cell type of each cluster was identified by known marker genes. For each cell type, we re‐ran the Seurat cluster workflow to identify cell subtypes.

### 4.8 Gene Set Score Analysis

The AddModuleScore function in Seurat was used to calculate module scores for gene expression programs in single cells. First, all the analyzed genes were binned based on the average expression, and the control genes were randomly selected from each bin. Then, the average expression value of the gene set was calculated at the single‐cell level minus the aggregated expression of the control gene set. Gene sets were obtained from the published paper (Saul et al. 2022) and the MSigDB database (https://www.gsea‐msigdb.org/gsea/msigdb/).

### 4.9 Analysis of the Interaction Between Cells

Cell–cell communication across distinct cell types was assessed using CellChat (v.1.4.0, R package). CellChat uses a gene expression matrix to predict the probability of cell‐to‐cell communication. This is achieved by combining the gene expression data with the existing database, including signaling molecules, receptors, and their associated cofactors. Cell–cell interactions were examined for each group in this work using the standard process. Normalized count data from each condition were utilized to generate a CellChat object, and the suggested preparation procedures were implemented for analyzing individual datasets using default values. The CellChatDB.human database was utilized for deducing cell–cell communication. All ligand‐receptor interaction categories from the database were included in the study. Communications with fewer than 10 cells were not considered.

### 4.10 Pseudotime Analysis

In this study, we used Slingshot (Street et al. 2018) for pseudotime analysis. Slingshot is a topology‐based pseudotime inference method that derives developmental trajectories from scRNA‐seq data. Initially, we applied principal component analysis (PCA) for dimensionality reduction of the data and then constructed a minimum spanning tree (MST) in the reduced dimensional space to connect all cell clusters. Slingshot then identifies the developmental starting point based on the MST structure and infers a pseudotime value for each cell. This approach allowed us to reconstruct the developmental pathways of cells in different states and identify potential differentiation processes.

### 4.11 Cell Culture and Treatment

Htori‐3.1 cells were transfected with *BMAL1*‐targeting siRNA (RIBOBIO) using Lipofectamine 3000 (Thermo, L3000015). Cells were exposed to 50 nM siRNAs for 6 h and then grown in RPMI1640 with 10% FBS for an additional 24 h. Htori‐3.1 cells were also infected with lentivirus expressing Cas9 and sgRNA targeting *BMAL1* (WZ Biosciences Inc.). Cells were incubated with the CRISPR‐Cas9 virus for 12 h, then replaced in RPMI1640 with 10% FBS for 24 h, and after 48 h of puromycin selection, single clones were selected and cells were collected for knockout validation using western blot. The siRNAs and sgRNA sequences are provided in Table S7. To induce cellular senescence, cells were exposed to etoposide (2 μM, MedChemExpress) for 24 h or LPS (1 μg/mL, TargetMol, USA) for 7 days. Subsequently, the cells were utilized for *RT–qPCR, Western blot analysis, and SA‐β‐gal staining*.

### 4.12 RNA‐Seq Library Preparation and Sequencing

Total RNA was extracted from 1 × 10^6^ cells with TRIzol Reagent (Invitrogen). After assessing the concentration of RNA with the Qubit TM dsDNA HS Assay Kit (Thermo), 1 μg per sample was constructed into libraries through the VAHTS Universal V6 RNA‐seq Library Prep Kit for MGI (Vazyme Biotech, NRM604‐01) following the manufacturer's manuals. The enrichment of fragments of interest was performed via VAHTSTM DNA Clean Beads (Vazyme Biotech, N411‐03). Sequencing data were generated on the DNBSEQ‐T7 platform with a 150‐bp paired‐end read length by MGI.

### 4.13 RNA‐Seq Data Processing

Raw RNA‐seq reads contaminated with adapters and reads with low‐quality bases were discarded to obtain the clean reads, and then clean reads were mapped against UCSC human reference hg38 using fastp (V0.20.1), and uniquely mapped reads were counted using HISAT2 (hisat2‐2.0.4). Gene expression levels were quantified with RPKM (reads per kilobase million). DEGs were identified with the R package DESeq2 (V1.260), and we calculated Benjamini & Hochberg FDR to obtain the statistical significance of DEGs. Genes were selected as DEGs only if the Fold Change > 2 and the *p*‐value < 0.01.

### 4.14 RNA Isolation and RT–qPCR

Total RNA was extracted with TRIzol Reagent (Invitrogen). After assessing the concentration of RNA with a NanoDrop 2000 ultra‐microspectrophotometer (Thermo), 2 μg total RNA was reversed as cDNA using a HiScript II Q RT SuperMix for qPCR (+gDNA wiper) kit. The selected candidate genes were validated by qPCR. Briefly, cDNA was synthesized with Maxima H Minus Reverse Transcriptase (Thermo Fisher Scientific, EP0751) in accordance with the manufacturer's instructions. Two‐step PCR was performed using SYBR Green PCR Master Mix (Applied Biosystems, 4344463) in accordance with the manufacturer's instructions on a LightCycler96 fluorescence sequence detection system (Roche). Gene expression was quantified relative to that of the housekeeping gene *GAPDH* and normalized to the control by the standard 2^−∆∆CT^ calculation. The primer sequences were as follows in Table S7.

### 4.15 SA‐β‐Gal Staining

We utilized a Senescence β‐Galactosidase Staining kit from Beyotime Biotechnology for SA‐β‐gal staining. Cells or thyroid tissue were rinsed with PBS and then placed in fixative solution for 30 min at room temperature. Cells were washed in PBS three times for 3 min each, then incubated in dye working solution overnight at 37°C without CO2. The following day, the dye working solution was disposed of and rinsed with PBS three times. The sections were viewed under the microscope. For quantitative analysis, five random fields of view were selected, and the percentage of SA‐β‐Gal‐positive cells in each field was calculated.

### 4.16 Western Blot

Cells were used to extract proteins. Following quantification with a BCA kit, 25 μg of protein per sample was utilized for Western blot analysis. Proteins were isolated using a 10% SDS‐PAGE gel and subsequently transferred to polyvinylidene fluoride (PVDF) membranes. Blots were blocked with 5% BSA for 2 h at room temperature, then treated with specific primary antibodies (BMAL1 (Novus Biologicals, NB100‐2288) 1:1000, GAPDH (Abways, AB0037) 1:20,000) overnight at 4°C. On the following day, the blots were reheated for one hour and then exposed to secondary antibodies for two hours. A ChemiDoc TMXRS+ system was finally utilized for image capture.

### 4.17 Immunohistochemistry

After deparaffinization and rehydration, antigen retrieval of sections was conducted using EDTA Antigen Repair Buffer (pH 9.0) at 100°C for 20 min. Then sections were washed in PBS three times for 5 min each. For IHC, the sections were incubated in 3% H_2_O_2_ for 30 min at room temperature. After blocking with 5% BSA for 30 min at 37°C, sections were incubated in primary antibodies (Table S8) at 4°C overnight. The next day, sections were washed in PBS three times and then incubated in secondary antibodies for 2 h at room temperature. Finally, sections were stained with diaminobenzidine (DAB) for 5 min and examined under the microscope.

The staining of CDKN1A or CDKN2A was assessed using the Remmele immunoreactive score (IRS) (Remmele and Stegner 1987) by multiplying the level of staining intensity (0–3 points: absent, weak, intermediate, and strong) with the percentage of positive cells (0–4 points: cutoffs: 0%, < 10%, 11%–50%, 51%–80%, and > 80%). The staining intensity was evaluated according to the following scale: negative (0 points), weakly positive (1 point), and positive (> 2 points).

### 4.18 Immunofluorescence

After deparaffinization and rehydration, antigen retrieval of sections was conducted using EDTA Antigen Repair Buffer (pH 9.0) at 100°C for 20 min. Then sections were washed in PBS three times for 5 min each. After blocking with 5% BSA for 30 min at 37°C, sections were incubated in primary antibodies (Table S8) at 4°C overnight. The next day, sections were washed in PBS three times and then incubated in a fluorescently labeled secondary antibody for 60 min at room temperature. After washing, the sections were counterstained with DAPI for 10 min, followed by mounting and examination under a fluorescence microscope.

### 4.19 ELISA Detection of Hormone Levels in Mouse Serum

Serum hormone levels of TSH and T4 were measured in young wild‐type mice, old wild‐type mice, and young mice with thyroid‐specific deletion of *Bmal1*. The measurements were performed using the mouse thyroxine (T4) ELISA Kit (Jiangsu Meimian Industrial Co. Ltd., MM‐0575M1) and Mouse Thyroid Stimulating Hormone (TSH) ELISA Kit (Jiangsu Meimian Industrial Co. Ltd., MM‐0564M2), following the manufacturer's instructions.

## Author Contributions

H.G. conceived and supervised the project. D.Z., B.S., and Q.Y. performed the experiments and conducted all the sample preparation for sequencing. D.Z. performed the data analysis; D.Z. and H.C. contributed to the experiments. D.Z. wrote the original manuscript. H.G. helped edit and revise the manuscript. All authors have read and approved the final manuscript.

## Disclosure

Materials Availability: All non‐commercial reagents used in this paper are available from the lead contact upon request.

Quantification and statistical analysis: The analysis software and quantification methodology that are specific to RNA‐seq and scRNA‐seq experiments are included under the relevant subsections of the Methods details section. Information regarding replicate numbers is provided in the figure legends. If error bars are used in figures, information about what error bars represent is also provided in the figure legend. If the degree of significance is provided in the figure legend, further details regarding the statistical test used are provided in the relevant subsections of the methods details that are specific to the analysis being performed. Statistical analyses and approximations were performed with GraphPad Prism 9 software (GraphPad). Statistical significance was analyzed with Student's *t*‐tests and two‐way ANOVA. A *p*‐value of less than 0.05 was considered statistically significant, *p < 0.05, **p < 0.01, ***p < 0.001, ****p < 0.0001. Data are presented as the upper, center, and lower lines indicate the 75% quantile +1.5 * interquartile range (IQR), 50% quantile, and 25% quantile −1.5 * IQR, respectively, which are indicated in the figure legends.

## Conflicts of Interest

The authors declare no conflicts of interest.

## Supporting information


**Figure S1.** Cell distribution by single‐cell RNA‐seq analysis of human thyroid in young, middle‐aged, and old groups. (A) UMAP plots displaying the major cell types in the young, middle‐aged, and old groups of the human thyroid. (B) UMAP plots displaying the samples, accessions, and states for the young, middle‐aged, and old groups in the human thyroid. (C) UMAP plots displaying the groups in the young, middle‐aged, and old groups of the human thyroid.

**Figure S2.** Cell type identification by single‐cell RNA‐seq analysis of human thyroid. (A) UMAP showing the expression levels of cell‐specific marker genes in human thyroid cell subpopulations. The color indicates the expression level. (B) The signature terms of cell‐specific marker genes in human thyroid cell subpopulations.

**Figure S3.** Shared gene analysis between aging‐related and thyroid disease genes with DEGs across cell types. (A) Heatmap showing the shared genes between hotspot genes of aging‐related genes in the GenAge database and DEGs of each cell type in each group. (B) Heatmap showing the shared genes between hotspot genes of aging‐related genes in the thyroid disease gene set and DEGs of each cell type in each group.

**Figure S4.** Expression patterns of different cell types during human thyroid aging. (A) Coefficient of variation (CV) analysis showing the transcriptional noise of different cell types in human thyroid. (B) Bar chart showing the number of upregulated and downregulated differentially expressed genes (DEGs) identified for each cell type in human thyroid between old and young groups (O/Y), middle‐aged and young groups (M/Y), and old and middle‐aged groups (O/M). (C) Venn diagrams showing the shared upregulated and downregulated DEGs between O/Y and the combination of O/M and M/Y. (D) Principal component analysis (PCA) in silico bulk (scRNA‐seq) data of human thyroid epithelial cells from each group. The group of each sample is annotated on the dot. (E) Bar chart showing the Euclidean distance between samples.

**Figure S5.** Age‐dependent changes in human thyroid function. (A) Gene expression levels of *TG*, *TPO*, *TSHR*, and *PAX8* in thyroid epithelial cells of young, middle‐aged, and old groups. (B) Immunohistochemical assay of protein expression levels of TG, TPO, TSHR, and PAX8 in thyroid tissues of young, middle‐aged, and old groups. Scale bar, 100 μm. (C) Gene expression levels of *TG*, *TPO*, *TSHR*, and *PAX8* in thyroid epithelial subtype cells of young, middle‐aged, and old groups.

**Figure S6.** Validation of cellular senescence in the human tissue. (A) Immunohistochemical analysis of CDKN2A protein expression levels in thyroid tissues from young and old groups, showing representative images (scale bar, 100 μm) and statistical results of the Remmele immunoreactive score (IRS). (B) Representative images of SA‐β‐gal staining are shown for the young and old groups. Scale bar, 100 μm.

**Figure S7.** Validation of BMAL1 knockdown and knockout efficiency. (A, B) siRNA‐mediated knockdown (A) and sgRNA‐mediated knockout (B) of BMAL1. Representative Western blot images showing BMAL1 expression levels. From left to right: uncropped membrane, cropped membrane, Western blot results for BMAL1, and GAPDH.

**Figure S8.** Generation and validation of thyroid‐specific Bmal1 conditional knockout mice. (A) Schematic diagram of the breeding strategy between *TPO‐Cre* transgenic mice and *Bmal1* ^*flox/flox*^ mice. (B) PCR genotyping results of offspring. (C) Immunofluorescence detection of Bmal1 expression level in mouse thyroid tissue. Scale bar, 100 μm.

**Figure S9.** Cell Type Identification by single‐cell RNA‐seq analysis of mouse thyroid. (A) UMAP plots showing the expression levels of marker genes in mouse thyroid. The color indicates the expression level. (B) Dot plot showing the gene expression signatures of marker genes corresponding to each cell type in the mouse thyroid. The dot size indicates the fraction of expressing cells, and the color indicates the expression level. (C) Gene expression levels of *Tg* and *Tshr* in thyroid epithelial cells of young wild‐type mice, old wild‐type mice, and thyroid‐specific deletion of *Bmal1* in young mice.

**Figure S10.** Validation of cellular senescence in the mouse tissue. (A) Immunohistochemical analysis of Cdkn2a protein expression levels in thyroid tissues from Young_wt, Old_wt, and Young_mut groups, showing representative images (scale bar, 100 μm) and statistical results of the Remmele immunoreactive score (IRS). (B) Representative images of SA‐β‐gal staining are shown for Young_wt, Old_wt, and Young_mut groups. Scale bar, 100 μm.

**Figure S11.** NF‐κB pathway activation in the human and mouse groups. (A) Representative immunofluorescence images of p‐p65 in thyroid tissues from Young_wt, Old_wt, and Young_mut groups. Scale bar, 100 μm. (B) Representative immunofluorescence images of p‐p65 in thyroid tissues from young and old groups. Scale bar, 100 μm.

**Figure S12.** LPS induces cellular senescence and NF‐κB without suppressing BMAL1 expression activation in HTori‐3.1 cells. (A) Representative images and quantitative analysis of SA‐β‐gal staining are shown for cell lines in control and LPS‐treated groups (1 μg/mL) in HTori‐3.1 cells. Scale bar, 100 μm. (B) Representative immunofluorescence images of p‐p65 in the control and LPS‐treated groups (1 μg/mL) in HTori‐3.1 cells. Scale bar, 100 μm. (C) Western blot analysis of BMAL1 and GAPDH in control and LPS‐treated groups (1 μg/mL) in HTori‐3.1 cells.

**Figure S13.** Patterns of cellular interactions during thyroid aging. (A–C) Heatmap showing pattern of ligand‐receptor interactions between cell types during thyroid aging.

**Table S1.** Sample information.

**Table S2.** Human cell type‐specific genes.

**Table S3.** The GO term of human cell‐type‐specific genes.

**Table S4.** SASP Geneset.

**Table S5.** Mouse major cell type‐specific genes.

**Table S6.** Cdkn1a^high^ different genes.

**Table S7.** Oligo sequences.

**Table S8.** Antibody list.

## Data Availability

The processed data reported in this paper and the raw data are available for download from the Gene Expression Omnibus (GEO) at GSE275233 and GSE275234. Any additional information required to reanalyze the data reported in this paper is available from the lead contact upon request.
