# Supplementary figures and images for: Circadian Gene BMAL1 Regulation of Cellular Senescence in Thyroid Aging
Source: Aging Cell. 2025 May 28;24(8):e70119. doi: 10.1111/acel.70119 (PMC12341809; doi:10.1111/acel.70119)

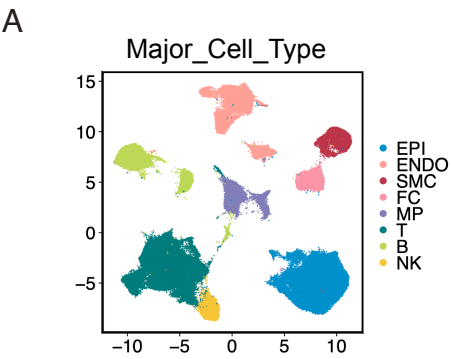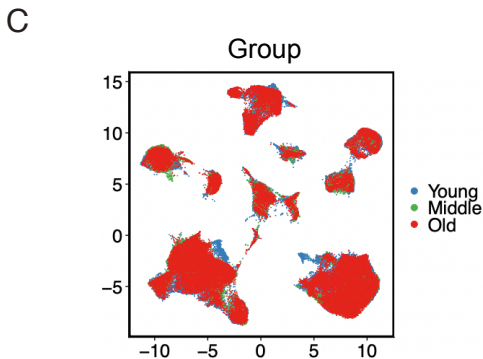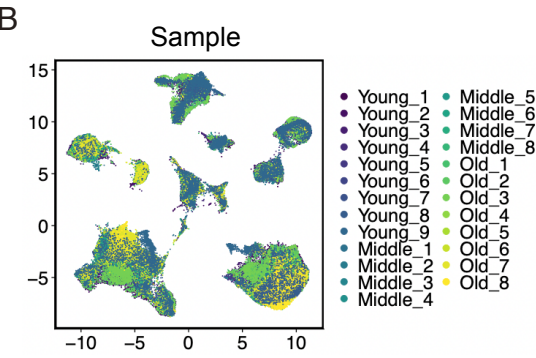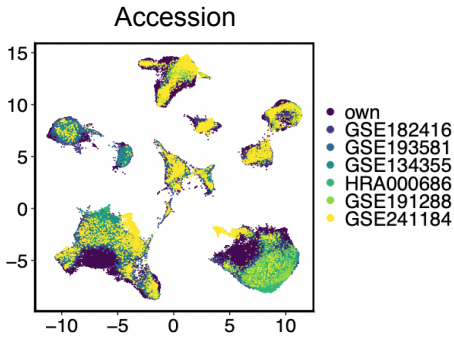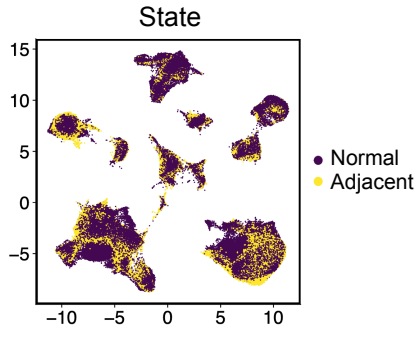

Supplement: Supplementary file 1 — Figure S1. Cell distribution by single‐cell RNA‐seq analysis of human thyroid in young, middle‐aged, and old groups. (A) UMAP plots displaying the major cell types in the young, middle‐aged, and old groups of the human thyroid. (B) UMAP plots displaying the samples, accessions, and states for the young, middle‐aged, and old groups in the human thyroid. (C) UMAP plots displaying the groups in the young, middle‐aged, and old groups of the human thyroid. [file ACEL-24-e70119-s020.pdf]

A

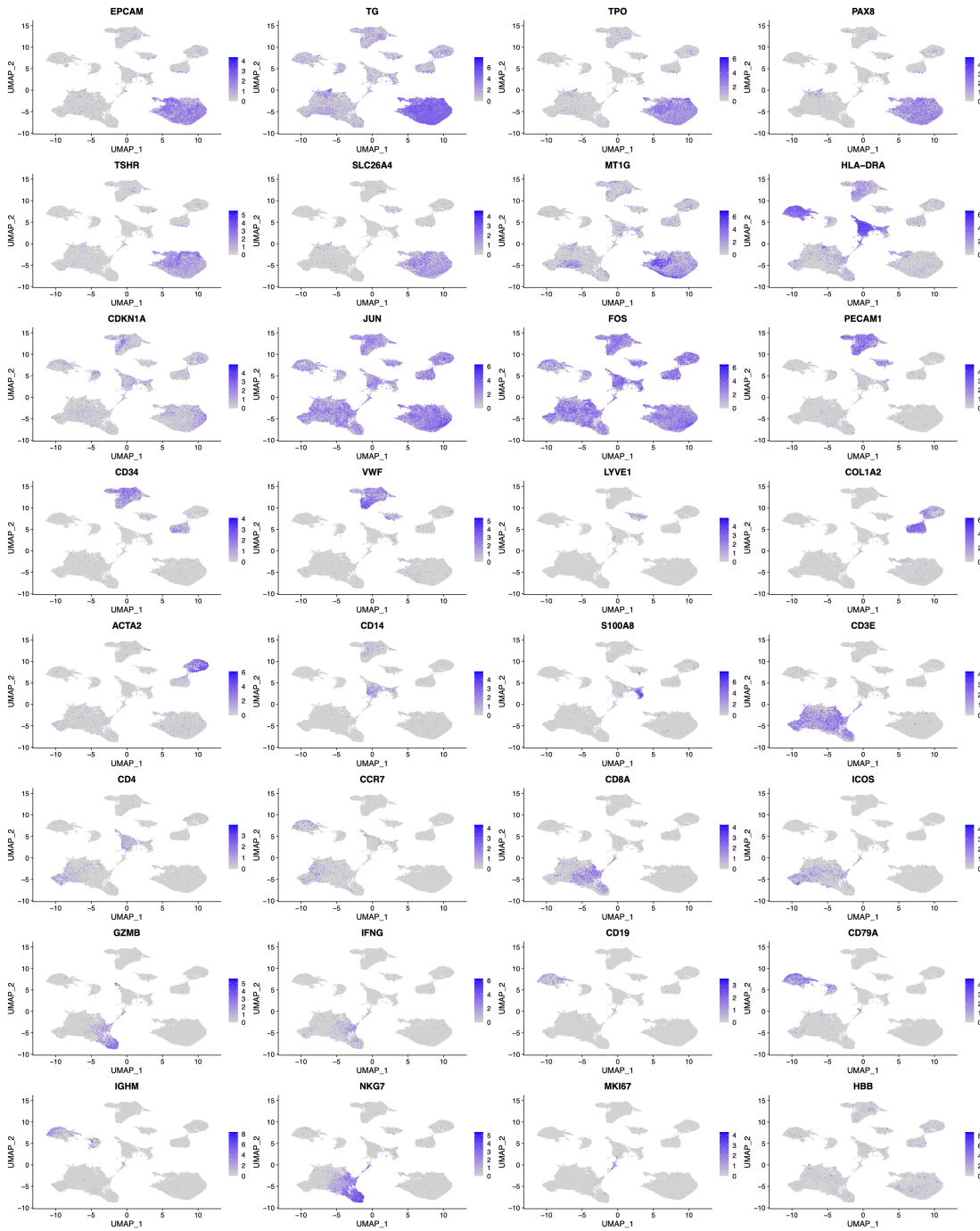

B

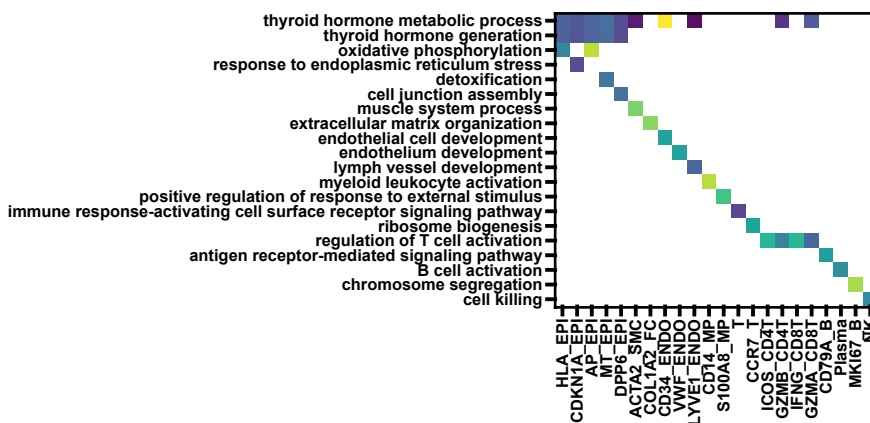

Supplement: Supplementary file 2 — Figure S2. Cell type identification by single‐cell RNA‐seq analysis of human thyroid. (A) UMAP showing the expression levels of cell‐specific marker genes in human thyroid cell subpopulations. The color indicates the expression level. (B) The signature terms of cell‐specific marker genes in human thyroid cell subpopulations. [file ACEL-24-e70119-s003.pdf]

A

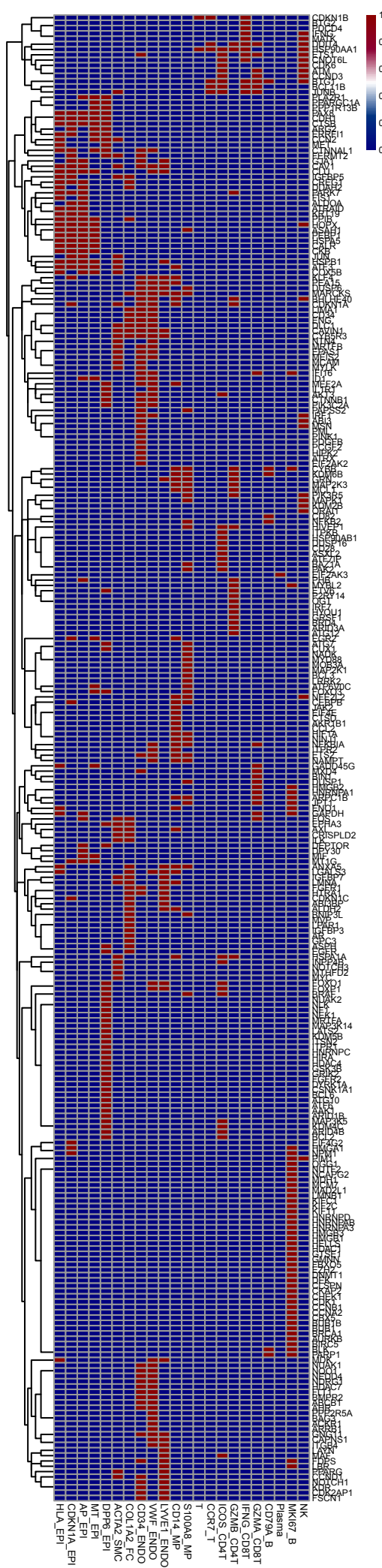

B

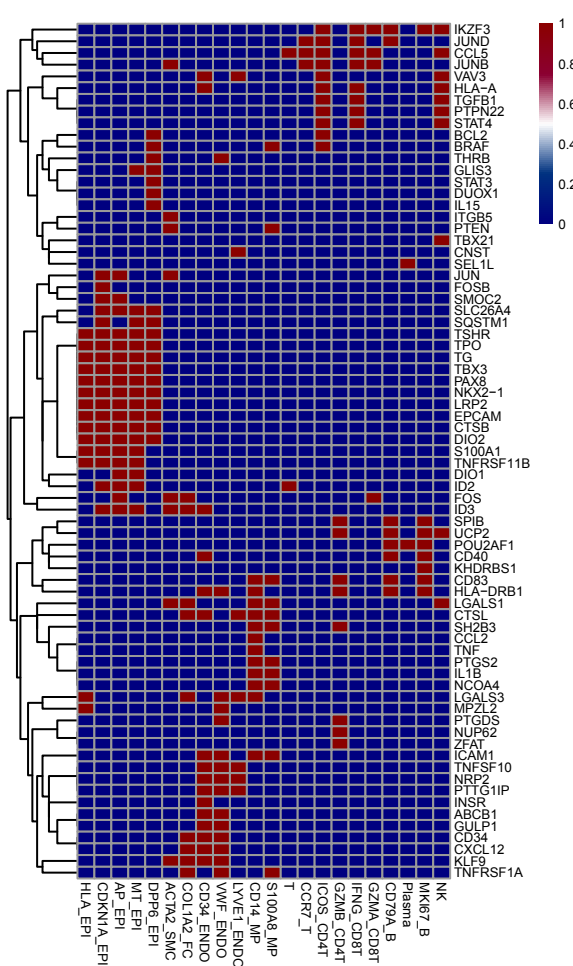

Supplement: Supplementary file 3 — Figure S3. Shared gene analysis between aging‐related and thyroid disease genes with DEGs across cell types. (A) Heatmap showing the shared genes between hotspot genes of aging‐related genes in the GenAge database and DEGs of each cell type in each group. (B) Heatmap showing the shared genes between hotspot genes of aging‐related genes in the thyroid disease gene set and DEGs of each cell type in each group. [file ACEL-24-e70119-s012.pdf]

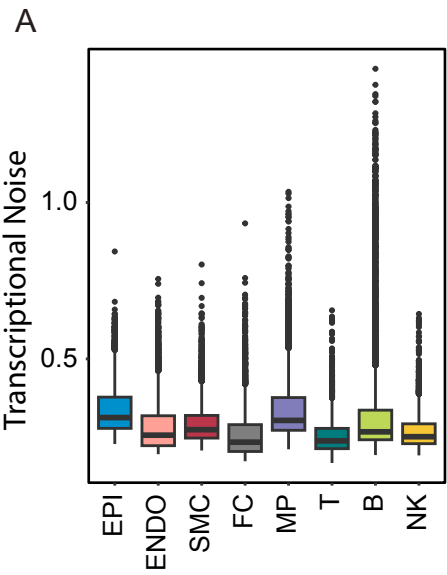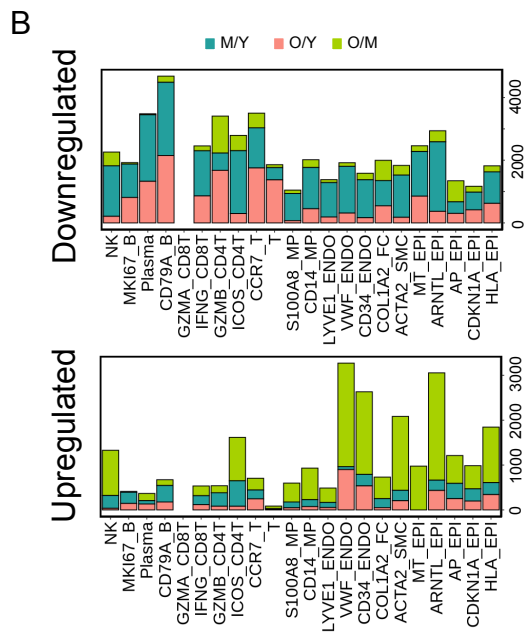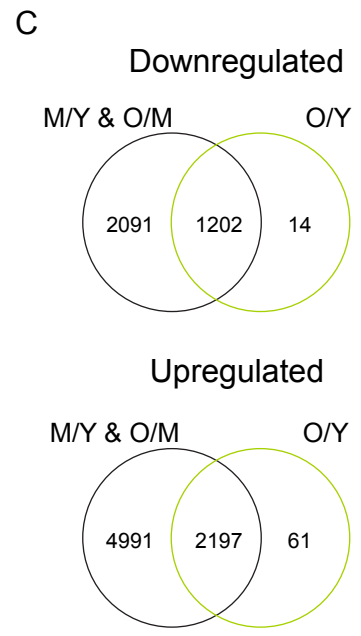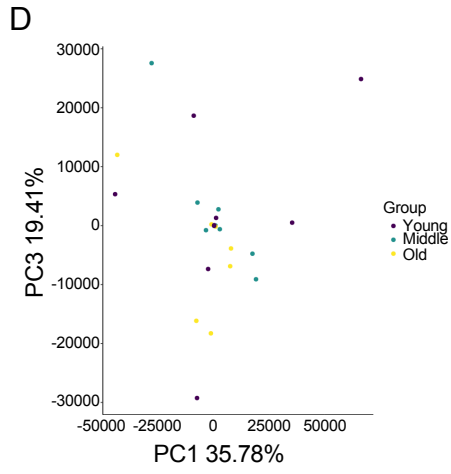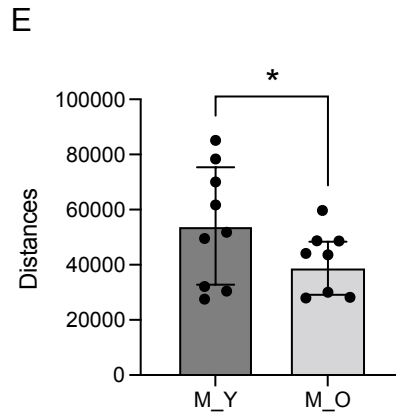

Supplement: Supplementary file 4 — Figure S4. Expression patterns of different cell types during human thyroid aging. (A) Coefficient of variation (CV) analysis showing the transcriptional noise of different cell types in human thyroid. (B) Bar chart showing the number of upregulated and downregulated differentially expressed genes (DEGs) identified for each cell type in human thyroid between old and young groups (O/Y), middle‐aged and young groups (M/Y), and old and middle‐aged groups (O/M). (C) Venn diagrams showing the shared upregulated and downregulated DEGs between O/Y and the combination of O/M and M/Y. (D) Principal component analysis (PCA) in silico bulk (scRNA‐seq) data of human thyroid epithelial cells from each group. The group of each sample is annotated on the dot. (E) Bar chart showing the Euclidean distance between samples. [file ACEL-24-e70119-s007.pdf]

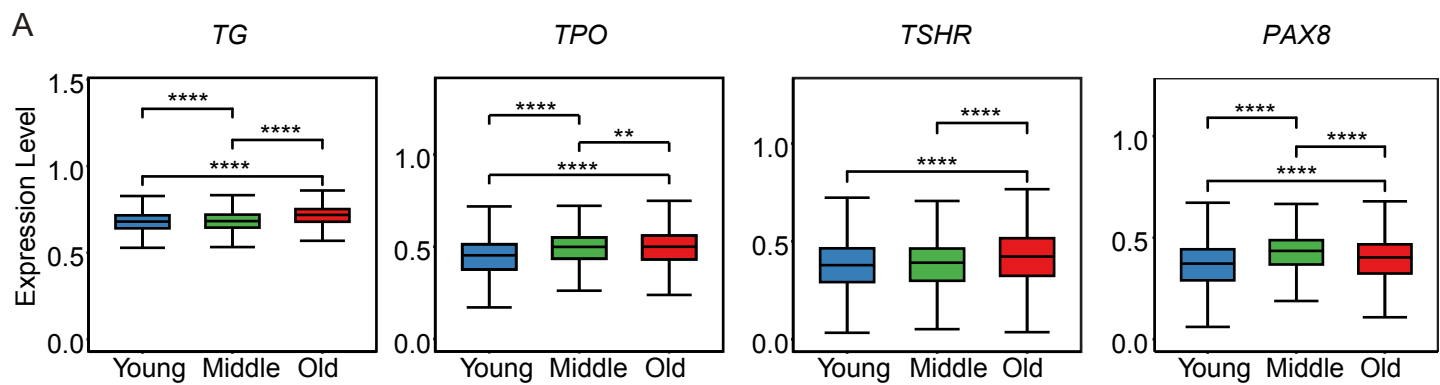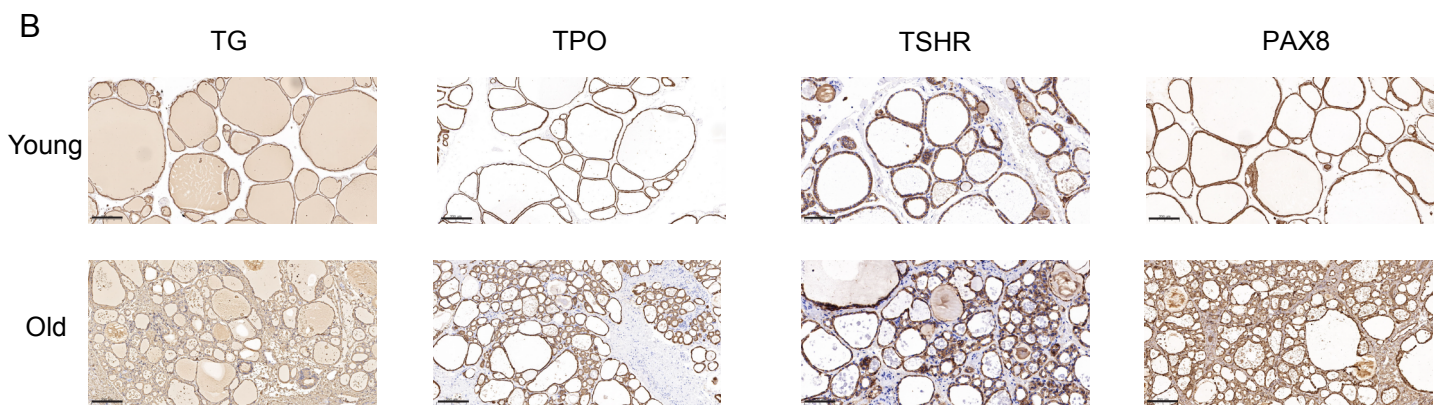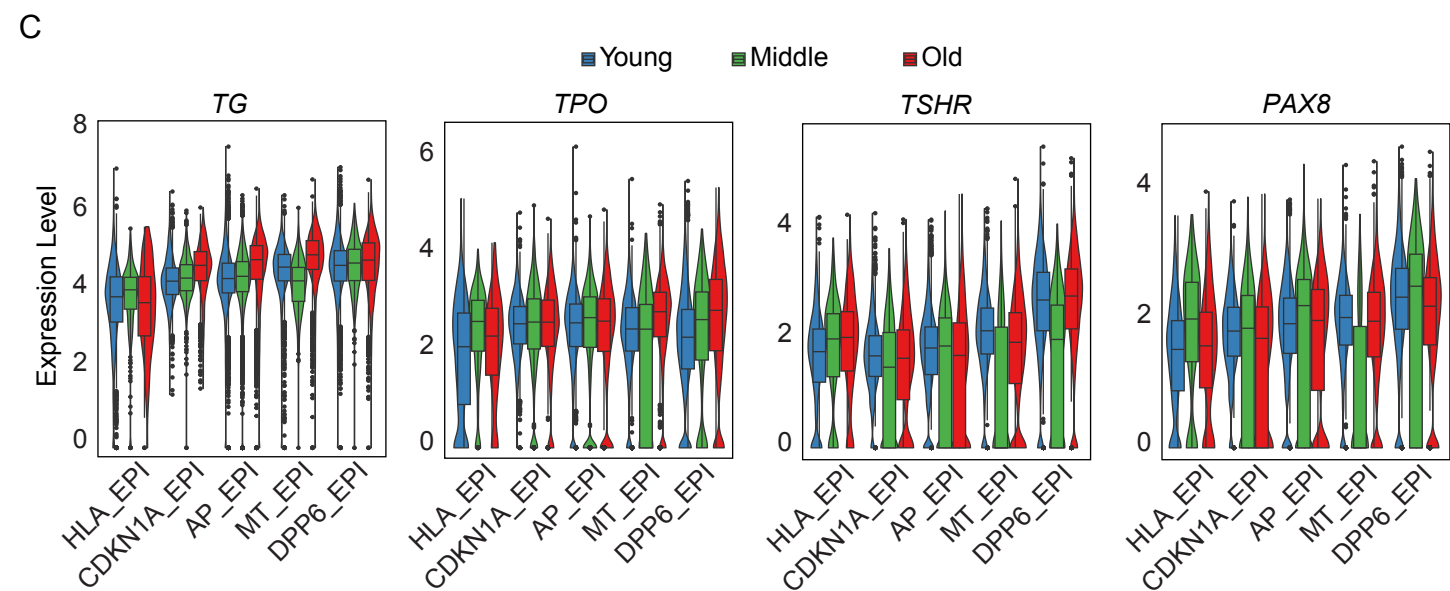

Supplement: Supplementary file 5 — Figure S5. Age‐dependent changes in human thyroid function. (A) Gene expression levels of TG, TPO, TSHR, and PAX8 in thyroid epithelial cells of young, middle‐aged, and old groups. (B) Immunohistochemical assay of protein expression levels of TG, TPO, TSHR, and PAX8 in thyroid tissues of young, middle‐aged, and old groups. Scale bar, 100 μm. (C) Gene expression levels of TG, TPO, TSHR, and PAX8 in thyroid epithelial subtype cells of young, middle‐aged, and old groups. [file ACEL-24-e70119-s019.pdf]

A

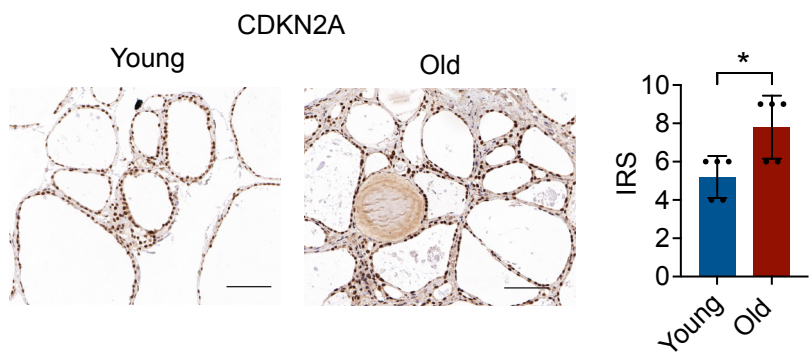

B

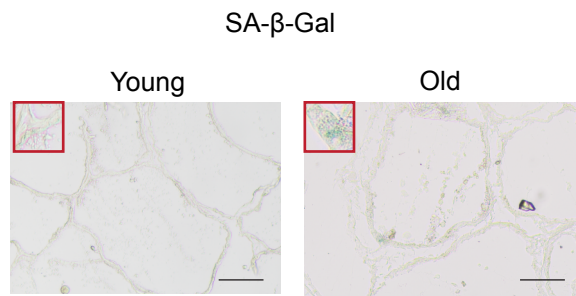

Supplement: Supplementary file 6 — Figure S6. Validation of cellular senescence in the human tissue. (A) Immunohistochemical analysis of CDKN2A protein expression levels in thyroid tissues from young and old groups, showing representative images (scale bar, 100 μm) and statistical results of the Remmele immunoreactive score (IRS). (B) Representative images of SA‐β‐gal staining are shown for the young and old groups. Scale bar, 100 μm. [file ACEL-24-e70119-s016.pdf]

A

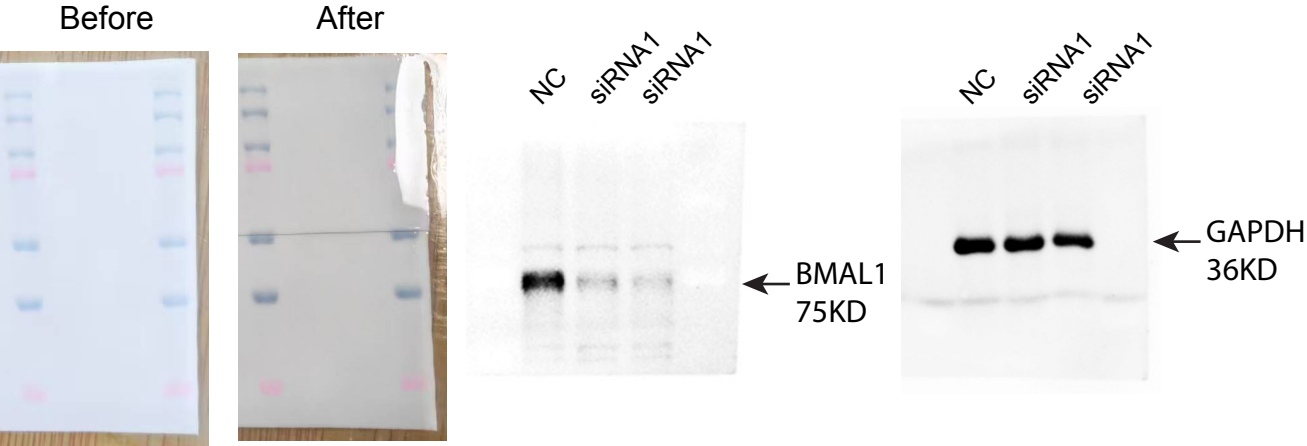

B

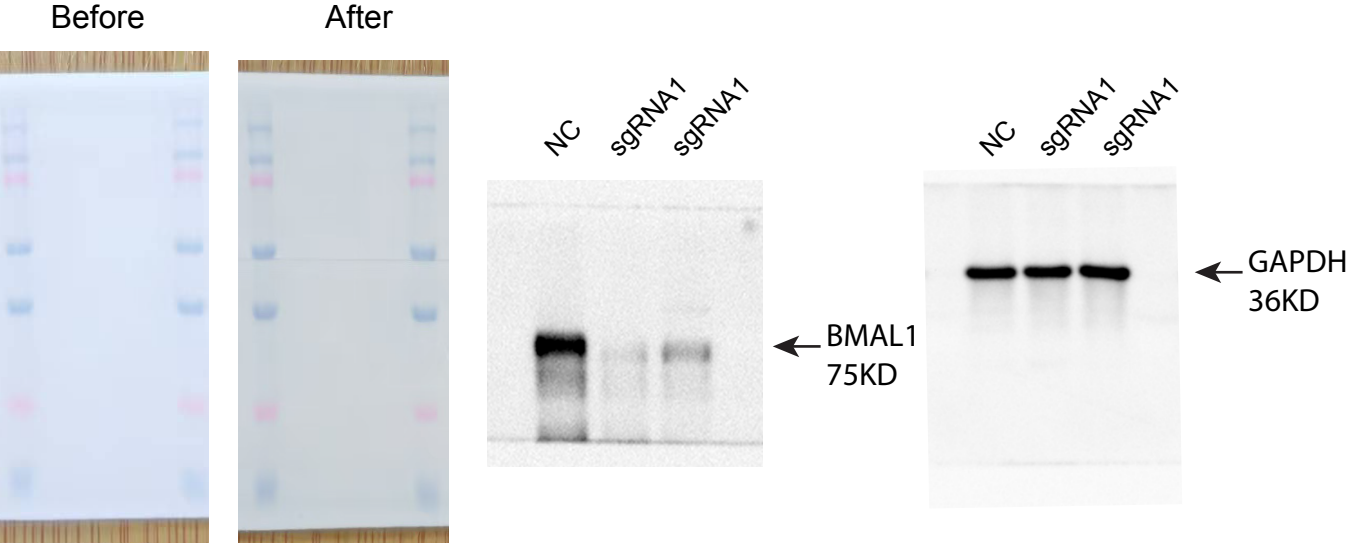

Supplement: Supplementary file 7 — Figure S7. Validation of BMAL1 knockdown and knockout efficiency. (A, B) siRNA‐mediated knockdown (A) and sgRNA‐mediated knockout (B) of BMAL1. Representative Western blot images showing BMAL1 expression levels. From left to right: uncropped membrane, cropped membrane, Western blot results for BMAL1, and GAPDH. [file ACEL-24-e70119-s009.pdf]

A

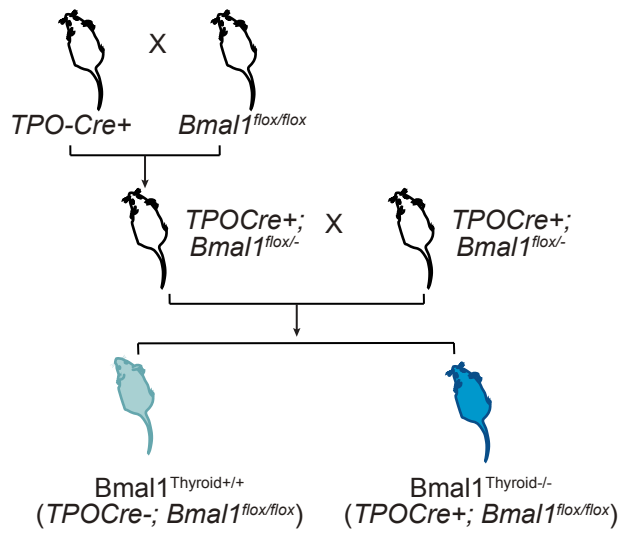

B

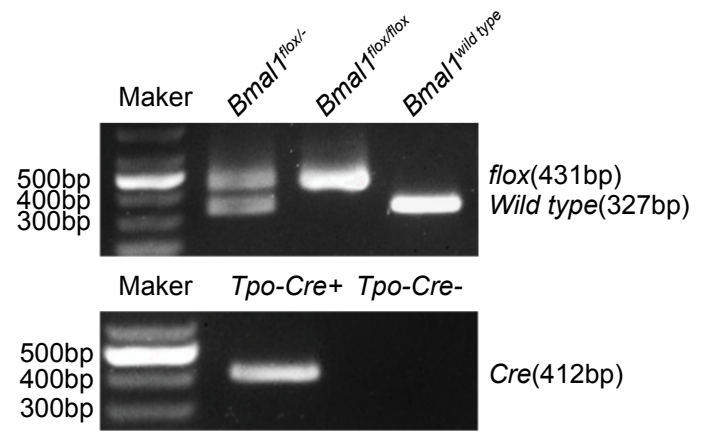

C

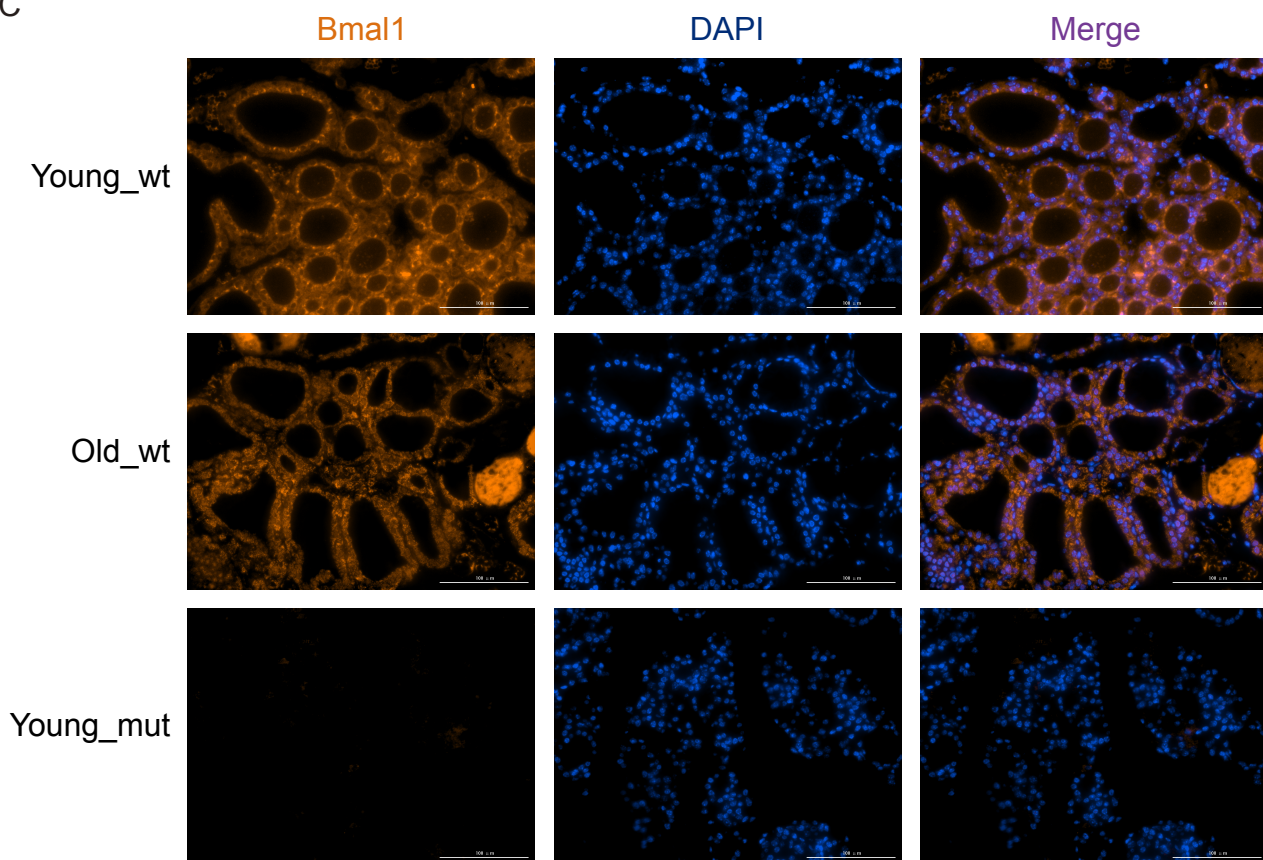

Supplement: Supplementary file 8 — Figure S8. Generation and validation of thyroid‐specific Bmal1 conditional knockout mice. (A) Schematic diagram of the breeding strategy between TPO‐Cre transgenic mice and Bmal1 flox/flox mice. (B) PCR genotyping results of offspring. (C) Immunofluorescence detection of Bmal1 expression level in mouse thyroid tissue. Scale bar, 100 μm. [file ACEL-24-e70119-s015.pdf]

A

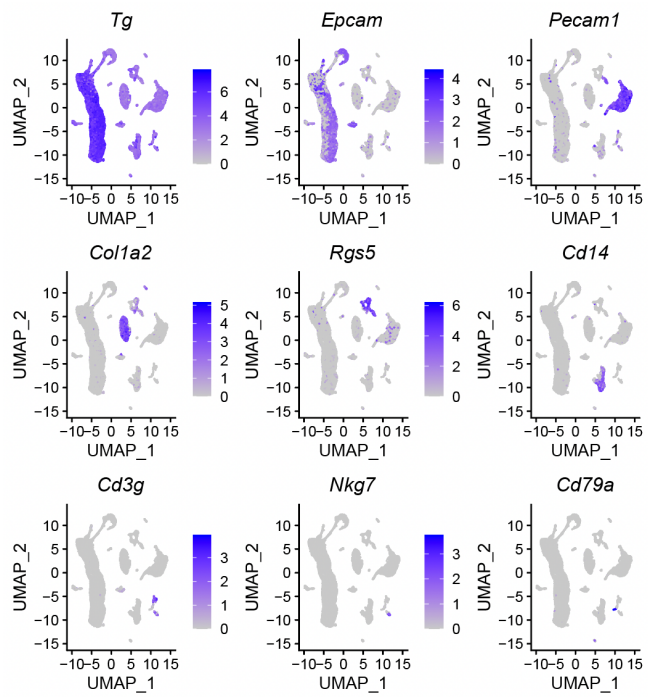

B

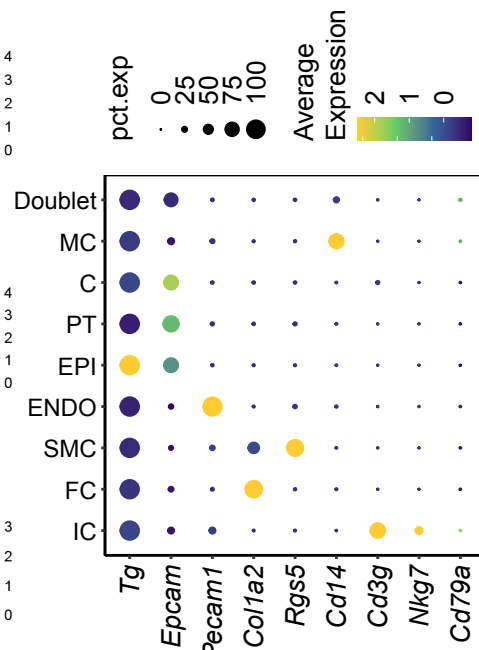

C

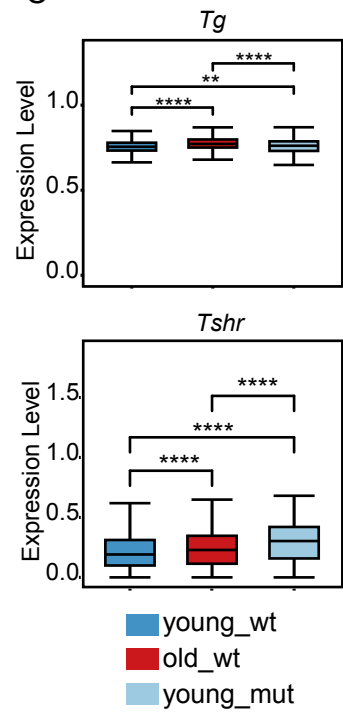

Supplement: Supplementary file 9 — Figure S9. Cell Type Identification by single‐cell RNA‐seq analysis of mouse thyroid. (A) UMAP plots showing the expression levels of marker genes in mouse thyroid. The color indicates the expression level. (B) Dot plot showing the gene expression signatures of marker genes corresponding to each cell type in the mouse thyroid. The dot size indicates the fraction of expressing cells, and the color indicates the expression level. (C) Gene expression levels of Tg and Tshr in thyroid epithelial cells of young wild‐type mice, old wild‐type mice, and thyroid‐specific deletion of Bmal1 in young mice. [file ACEL-24-e70119-s021.pdf]

A

Cdkn2a

Young\_wt

Old\_wt

Young\_mut

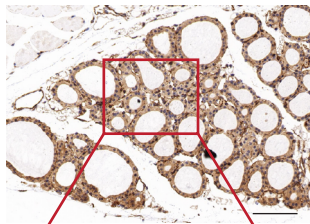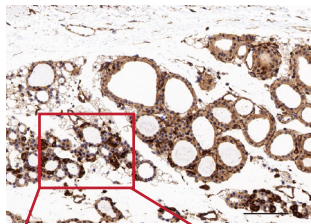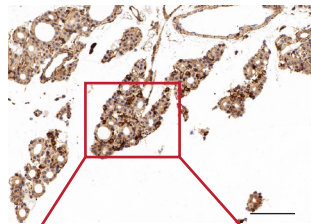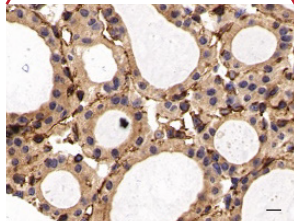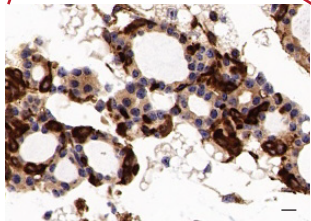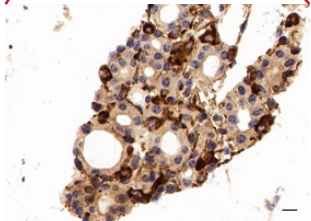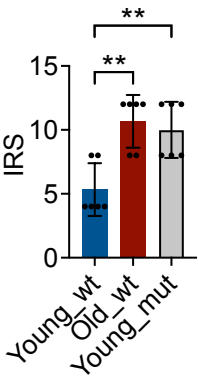

B

SA-β-Gal

Young\_wt

Old\_wt

Young\_mut

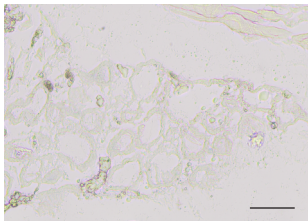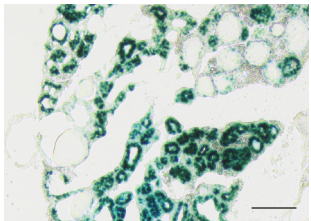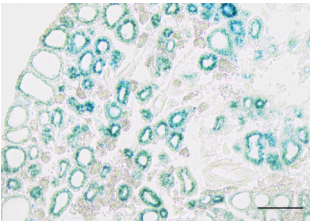

Supplement: Supplementary file 10 — Figure S10. Validation of cellular senescence in the mouse tissue. (A) Immunohistochemical analysis of Cdkn2a protein expression levels in thyroid tissues from Young_wt, Old_wt, and Young_mut groups, showing representative images (scale bar, 100 μm) and statistical results of the Remmele immunoreactive score (IRS). (B) Representative images of SA‐β‐gal staining are shown for Young_wt, Old_wt, and Young_mut groups. Scale bar, 100 μm. [file ACEL-24-e70119-s008.pdf]

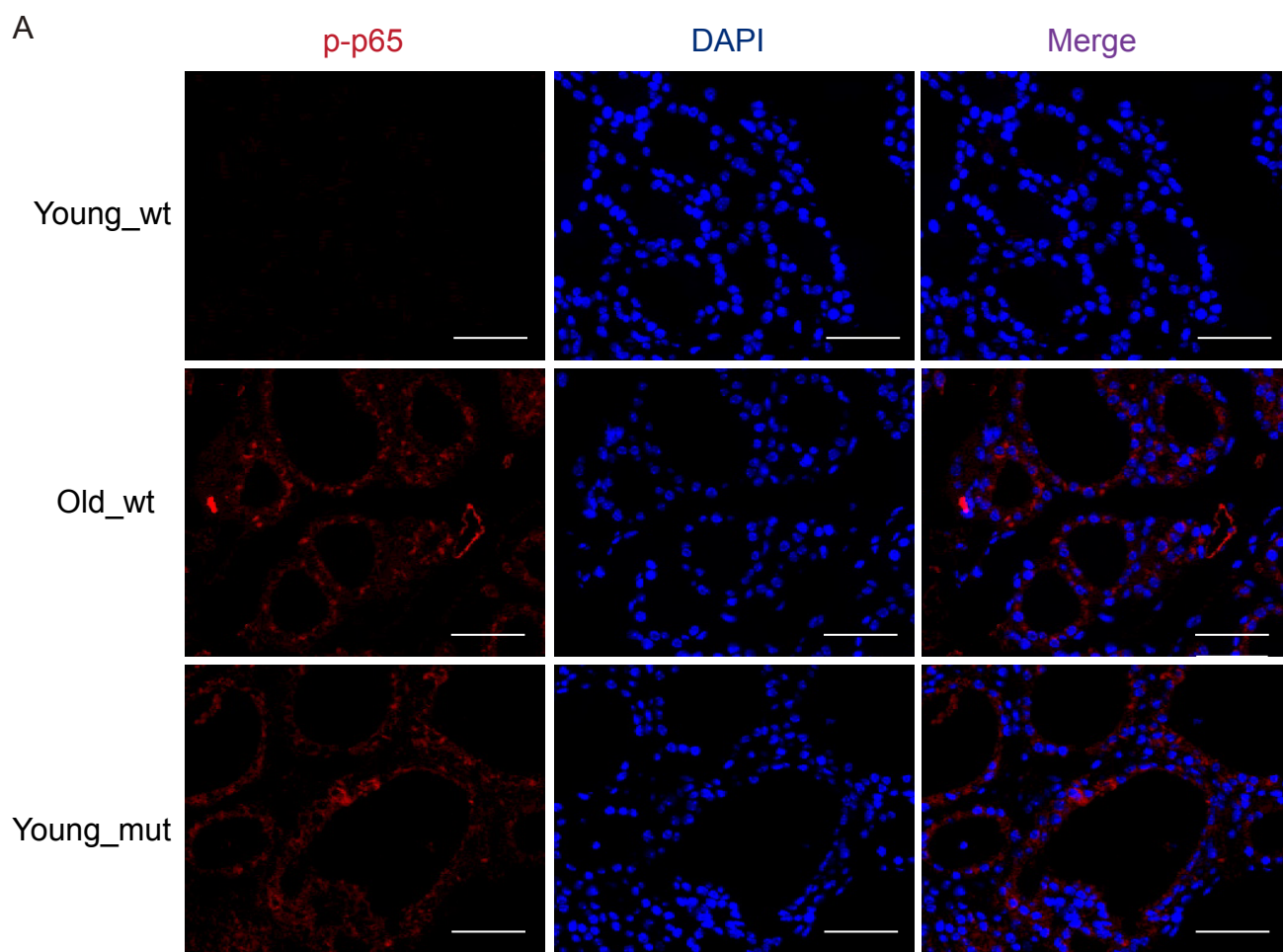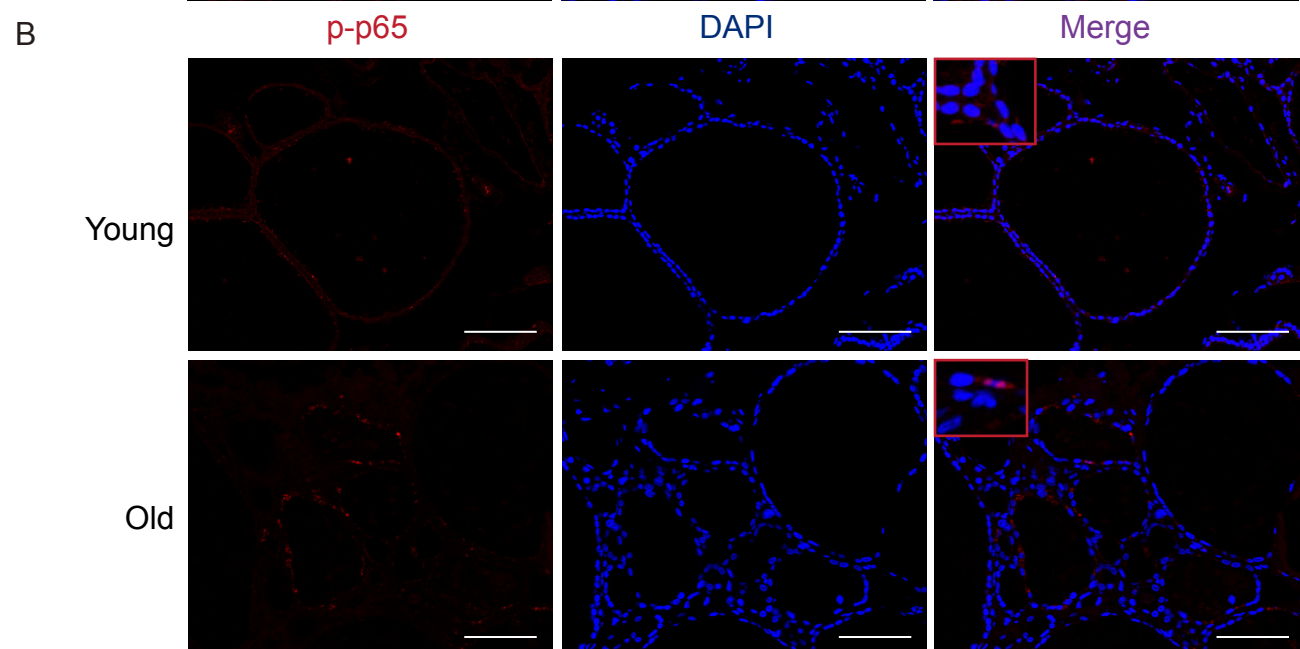

Supplement: Supplementary file 11 — Figure S11. NF‐κB pathway activation in the human and mouse groups. (A) Representative immunofluorescence images of p‐p65 in thyroid tissues from Young_wt, Old_wt, and Young_mut groups. Scale bar, 100 μm. (B) Representative immunofluorescence images of p‐p65 in thyroid tissues from young and old groups. Scale bar, 100 μm. [file ACEL-24-e70119-s017.pdf]

A

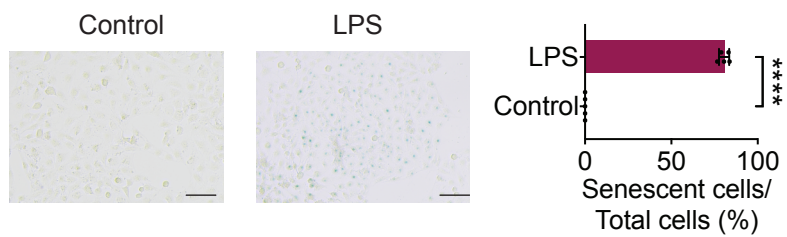

B

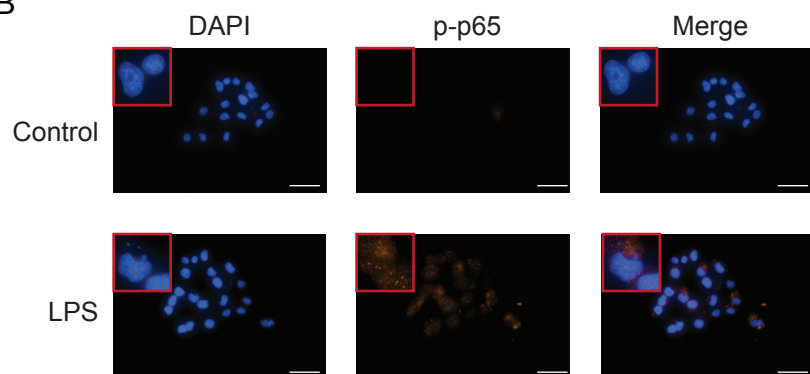

C

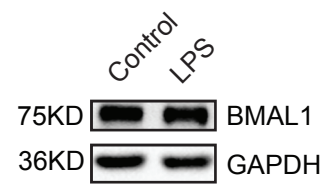

Supplement: Supplementary file 12 — Figure S12. LPS induces cellular senescence and NF‐κB without suppressing BMAL1 expression activation in HTori‐3.1 cells. (A) Representative images and quantitative analysis of SA‐β‐gal staining are shown for cell lines in control and LPS‐treated groups (1 μg/mL) in HTori‐3.1 cells. Scale bar, 100 μm. (B) Representative immunofluorescence images of p‐p65 in the control and LPS‐treated groups (1 μg/mL) in HTori‐3.1 cells. Scale bar, 100 μm. (C) Western blot analysis of BMAL1 and GAPDH in control and LPS‐treated groups (1 μg/mL) in HTori‐3.1 cells. [file ACEL-24-e70119-s014.pdf]
