# Supplementary material for: Circadian Gene BMAL1 Regulation of Cellular Senescence in Thyroid Aging
Source: Aging Cell. 2025 May 28;24(8):e70119. doi: 10.1111/acel.70119 (PMC12341809; doi:10.1111/acel.70119)
Supplement: Supplementary file 13 — Figure S13. Patterns of cellular interactions during thyroid aging. (A–C) Heatmap showing pattern of ligand‐receptor interactions between cell types during thyroid aging. [file ACEL-24-e70119-s006.pdf]

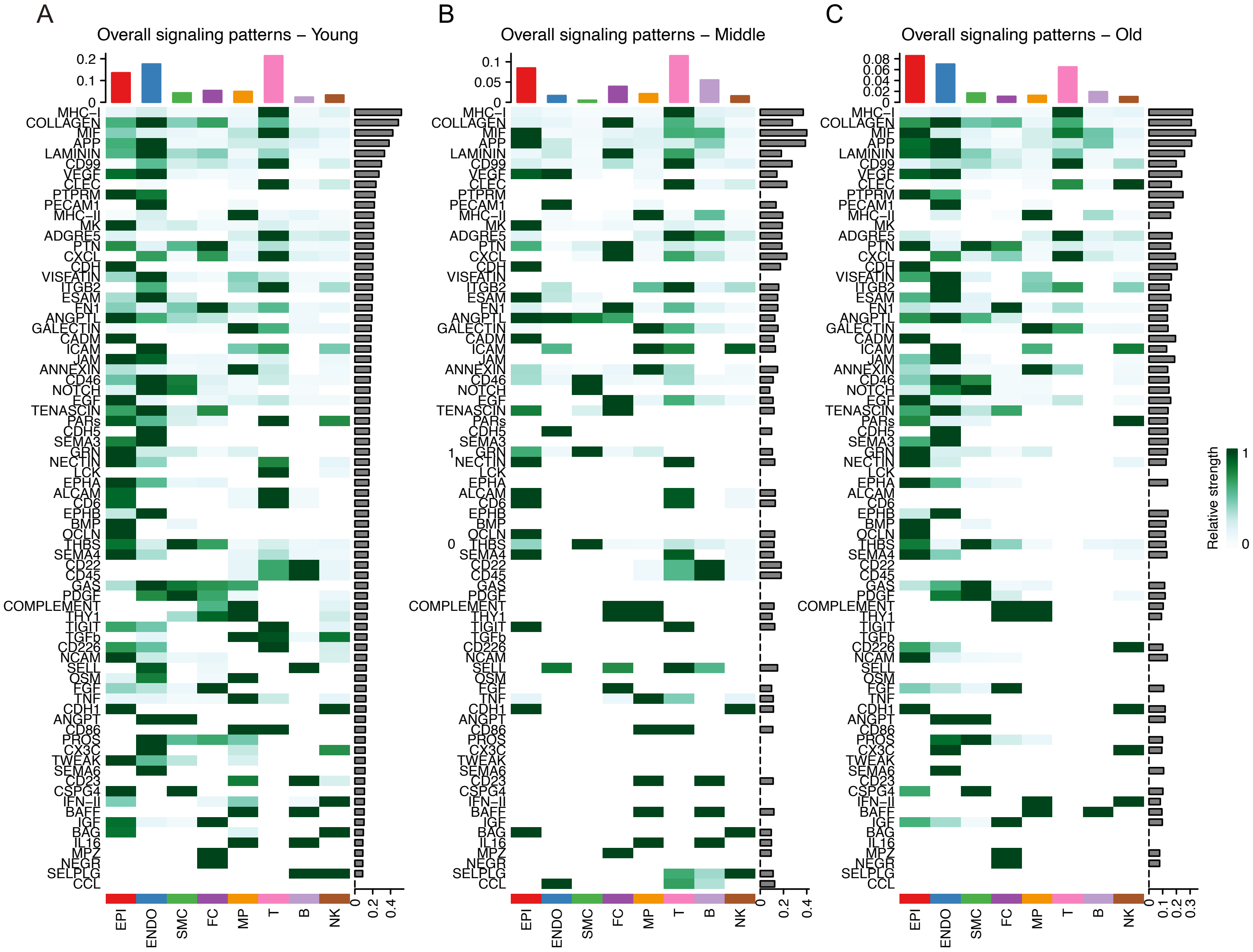
A
Overall signaling patterns – Young
0.2
0.1
0
MHC-I
COLLAGEN
MIF
APP
LAMININ
CD99
VEGF
CLEC
PTPRM
PECAM1
MHC-II
MK
ADGRE5
PTN
CXCL
CDH
VISFATIN
ITGB2
ESAM
FN1
ANGPTL
GALECTIN
CADM
ICAM
JAM
ANNEXIN
CD46
NOTCH
EGF
TENASCIN
PARs
CDH5
SEMA3
GRN
NECTIN
LCK
EPHA
ALCAM
CD6
EPHB
BMP
OCLN
THBS
SEMA4
CD22
CD45
GAS
PDGF
COMPLEMENT
THY1
TIGIT
TGFb
CD226
NCAM
SELL
OSM
FGF
TNF
CDH1
ANGPT
CD86
PROS
CX3C
TWEAK
SEMA6
CD23
CSPG4
IFN-II
BAFF
IGF
BAG
IL16
MPZ
NEGR
SELPLG
CCL
EPI
ENDO
SMC
FC
MP
T
B
NK
0
0.2
0.4
B
Overall signaling patterns – Middle
0.1
0.05
0
1
0
EPI
ENDO
SMC
FC
MP
T
B
NK
0
0.2
0.4
C
Overall signaling patterns – Old
0.08
0.06
0.04
0.02
0
EPI
ENDO
SMC
FC
MP
T
B
NK
0
0.1
0.2
0.3
Relative strength
1
0
